# Intrinsic Connectivity Networks of Glutamate-Mediated Antidepressant Response: A Neuroimaging Review

**DOI:** 10.3389/fpsyt.2022.864902

**Published:** 2022-05-26

**Authors:** Ilya Demchenko, Vanessa K. Tassone, Sidney H. Kennedy, Katharine Dunlop, Venkat Bhat

**Affiliations:** ^1^Interventional Psychiatry Program, Mental Health and Addictions Service, St. Michael's Hospital, Unity Health Toronto, Toronto, ON, Canada; ^2^Center for Depression and Suicide Studies, St. Michael's Hospital, Unity Health Toronto, Toronto, ON, Canada; ^3^Keenan Research Center for Biomedical Science, St. Michael's Hospital, Unity Health Toronto, Toronto, ON, Canada; ^4^Department of Psychiatry, Faculty of Medicine, University of Toronto, Toronto, ON, Canada

**Keywords:** major depressive disorder, glutamate, intrinsic connectivity networks, biomarkers, connectome, functional neuroimaging, ketamine, antidepressive agents

## Abstract

Conventional monoamine-based pharmacotherapy, considered the first-line treatment for major depressive disorder (MDD), has several challenges, including high rates of non-response. To address these challenges, preclinical and clinical studies have sought to characterize antidepressant response through monoamine-independent mechanisms. One striking example is glutamate, the brain's foremost excitatory neurotransmitter: since the 1990s, studies have consistently reported altered levels of glutamate in MDD, as well as antidepressant effects following molecular targeting of glutamatergic receptors. Therapeutically, this has led to advances in the discovery, testing, and clinical application of a wide array of glutamatergic agents, particularly ketamine. Notably, ketamine has been demonstrated to rapidly improve mood symptoms, unlike monoamine-based interventions, and the neurobiological basis behind this rapid antidepressant response is under active investigation. Advances in brain imaging techniques, including functional magnetic resonance imaging, magnetic resonance spectroscopy, and positron emission tomography, enable the identification of the brain network-based characteristics distinguishing rapid glutamatergic modulation from the effect of slow-acting conventional monoamine-based pharmacology. Here, we review brain imaging studies that examine brain connectivity features associated with rapid antidepressant response in MDD patients treated with glutamatergic pharmacotherapies in contrast with patients treated with slow-acting monoamine-based treatments. Trends in recent brain imaging literature suggest that the activity of brain regions is organized into coherent functionally distinct networks, termed intrinsic connectivity networks (ICNs). We provide an overview of major ICNs implicated in depression and explore how treatment response following glutamatergic modulation alters functional connectivity of limbic, cognitive, and executive nodes within ICNs, with well-characterized anti-anhedonic effects and the enhancement of “top-down” executive control. Alterations within and between the core ICNs could potentially exert downstream effects on the nodes within other brain networks of relevance to MDD that are structurally and functionally interconnected through glutamatergic synapses. Understanding similarities and differences in brain ICNs features underlying treatment response will positively impact the trajectory and outcomes for adults suffering from MDD and will facilitate the development of biomarkers to enable glutamate-based precision therapeutics.

## Introduction

Approximately 35% of patients with Major Depressive Disorder (MDD), which ranked among the top 25 leading causes of disease burden worldwide in 2019, do not respond to two or more different antidepressant medications and meet the criteria for Treatment-Resistant Depression (TRD) (1). TRD is associated with a high proportion of physical and psychiatric comorbidities, long-lasting functional impairment, and increased suicide risk (2–4). To find effective therapeutic approaches, MDD has been investigated as a disease of monoamine deficiency over the last century, which coincided with an explosion of pharmaceutical agents targeting the system (5, 6). Under the monoamine hypothesis, the antidepressant response was thought to be achieved by increasing the levels of monoamine neurotransmitters serotonin, norepinephrine, and dopamine in the synaptic cleft *via* the targeting of the molecular mechanisms of reuptake inhibition and enzymatic blockade. However, in the following years, several studies have cast doubt on the validity of low monoamine levels as an underlying all-or-nothing principle behind the etiology of MDD (7–10). Furthermore, the heterogeneity of response to monoaminergic antidepressants, low remission rates, and increased treatment refractoriness remain unmet challenges that have warranted the search for alternative treatment options with a rapid onset of action (11, 12).

In the early 1990s, the momentous discovery that N-methyl-D-aspartate receptor (NMDA-R) antagonists, notably ketamine, led to rapid improvements in depressive symptoms in rodents (13) and humans (14) served as an impetus for proposing the glutamate hypothesis of depression. Supplemented with early evidence of significantly higher plasma glutamate levels in patients with mood disorders (15), this etiological framework postulates that alterations in glutamate release, clearance, and metabolism lead to sustained accumulation of glutamate in cortical and limbic brain areas that regulate emotions, cognition, and behavior, thereby promoting a depressive state (16, 17). According to this theory, altered levels of synaptic and extrasynaptic glutamate transmission would result in impaired synaptic connectivity in these regions, manifested by decreased synaptogenesis, excitation-inhibition imbalance, neuronal loss and atrophy, and deficits in the inhibitory fine-tuning (18). This hypothesis has shifted drug discovery efforts toward identifying and investigating the properties of novel pharmacological agents that target the glutamatergic system, establishing a new paradigm in the research and treatment of MDD (Table 1).

**Table 1:** Glutamate-mediating drug candidates for major depressive disorder.

**Glutamatergic compound**	**Clinical trial identifier example**	**Phase**	**Status**
**Non-selective NMDA-R antagonists**			
Ketamine (PMI-100, PMI-150, R-107, SHX-001, SLS-002, TUR-002)	NCT02544607	IV	Completed
Esketamine (PGI-061)	NCT04829318	IV	Active
Dextromethorphan	NCT04226352	I–II	Active
Dextromethorphan/quinidine (AVP-786, CTP-786)	NCT02153502	II	Completed
Dextromethorphan/bupropion (AXS-05)	NCT04019704	III	Completed
Memantine	NCT00344682	IV	Completed
Nitrous oxide	NCT03283670	II	Completed
Lanicemine (AZD-6765)	NCT01482221	II	Completed
Riluzole	NCT01204918	II	Completed
Dextromethadone (REL-1017)	NCT04855760	III	Active
**NR2B-selective NMDA-R antagonists**			
EVT-101 (ENS-101)	NCT01128452	II	Terminated
Traxoprodil (CP-101,606)	NCT00163059	II	Completed
Rislenemdaz (MK-0657, CERC-301)	NCT00472576	I	Completed
MIJ821	NCT04722666	II	Active
**Glycine site partial NMDA-R agonists**			
Rapastinel (GLYX-13)	NCT01684163	II	Completed
Apimostinel (NRX-1074)	NCT02067793	II	Completed
D-cycloserine	NCT00408031	II	Completed
**Glycine site partial NMDA-R antagonists**			
4-chlorokynurenine (AV-101)	NCT02484456	II	Completed
**AMPA-R modulators**			
Farampator (CX-691, ORG-24448, SCH-900460)	NCT00113022	II	Terminated
2R,6R-hydroxynorketamine	NCT04711005	I	Active
TAK-653 (NBI-1065845)	NCT03312894	II	Withdrawn
Arketamine (PCN-10, HR-071603)	NCT04108234	I	Active
Diazoxide	NCT02049385	I-II	Terminated
**mGluR modulators**			
Decoglurant (RO4995819)	NCT01457677	II	Completed
Basimglurant (RO4917523)	NCT00809562	II	Completed
TP0473292 (TS-161)	NCT03919409	I	Completed
N-Acetylcysteine	NCT04005053	II	Active
BCI-838	NCT01548703	I	Completed
BCI-632	NCT01546051	I	Completed
BCI-1038	NCT01546051	I	Completed
BCI-1206	NCT01546051	I	Completed
BCI-1283	NCT01546051	I	Completed

*AMPA-R, α-amino-3-hydroxy-5-methyl-4-isoxazolepropionic acid receptor; mGluR, metabotropic glutamate receptor; NMDA-R, N-methyl-D-aspartate receptor*.

In the clinical setting, significant efforts have been undertaken toward establishing the efficacy, feasibility, and safety of glutamatergic interventions for MDD and TRD, with intravenous ketamine and intranasal s-ketamine researched the most extensively (19). However, the attempts to identify biological predictors of rapid antidepressant response to glutamate-mediating interventions have not achieved a clinically meaningful predictive value at an individual level. To date, glutamatergic clinical research is mainly hypothesis-driven, as opposed to being data-driven, and the field is missing translational predictive preclinical models. The findings concerning the effects of glutamate-mediating compounds on the human brain connectome remain unconsolidated. This makes the identification of robust and reproducible biomarkers of treatment effects and rapid antidepressant response challenging, thus limiting the application of these findings to the real-world psychiatric setting, where the heterogeneity of symptoms and treatment response as well as the presence of physical and psychiatric comorbidities are significant factors.

Motivated by these limitations, recent studies have sought to employ new methodologies to advance our understanding of individual differences that predict and characterize antidepressant response. Research has largely focused on techniques that provide quantifiable metrics of biological structure and function, such as multi-omics (transcriptomics, proteomics, metabolomics), neuroimaging, and network pathway analysis (19). Neuroimaging, in particular, provides robust and reproducible models of the functional neuroanatomy and the brain's network architecture, which can serve as predictive correlates of clinical and functional outcomes (20). One of the major advances in the field of neuroimaging is the demonstration that the activity of brain regions is organized into coherent networks that are functionally distinct (21). These networks, termed intrinsic connectivity networks (ICNs), represent coupled brain regions correlated over time in spontaneous or task-evoked activity fluctuations (22). ICNs are associated with human cognition and behavior that can be studied at rest or using neuropsychological paradigms (23). They are highly replicable (24–26) and are thought to be constrained by brain anatomy, sufficiently reflecting the structural topology of the brain (27–29). Studying functional alterations in ICNs specific to MDD and TRD can elucidate the heterogeneity of symptom manifestation and treatment response, serving as predictive biomarkers of resistance to treatment and impacting clinical and functional outcomes (30–32).

In this article, we aim to summarize the key advances in our understanding of how glutamate-mediating interventions modulate ICNs of the human brain. First, we provide a brief overview of the functional architecture of human glutamatergic networks and how alterations in their activity and connectivity features could overlap with those previously described in the context of ICNs pertinent to MDD. Next, we review brain imaging studies across modalities that report ICN activity and connectivity alterations predicting or characterizing rapid antidepressant response to glutamate-mediating interventions. Finally, we discuss how these ICN biomarkers contrast with those of slow-active monoamine-based treatments for MDD and provide commentary on the direction for the next generation of neuroimaging biomarker studies for glutamatergic treatments.

## Overview of Glutamatergic Neurocircuitry

Glutamate is a major excitatory neurotransmitter in the brain, and ~80% of all neocortical synapses are glutamatergic (33). Upon release in the synaptic cleft, glutamate triggers changes in the conduction of action potential, neurotrophic function, and apoptosis pathways by binding to ionotropic NMDA-Rs, α-amino-3-hydroxy-5-methyl-4-isoxazolepropionic acid receptors (AMPA-Rs), and kainate receptors on the postsynaptic membrane (34). In parallel, glutamate binds to G-protein-coupled metabotropic glutamate receptors (mGluRs), mediating changes in cellular processes that are regulated by second messenger molecular cascades (34). At the systems level, these molecular alterations translate into structural and functional changes within glutamatergic neurocircuitry, which could contribute to psychopathology either directly, *via* the recruitment of the core brain areas and ICNs receiving glutamatergic innervation, or indirectly, *via* the modulation of monoamine, gamma-aminobutyric acid (GABA), and other neurotransmitter circuits.

**Figure 1:**
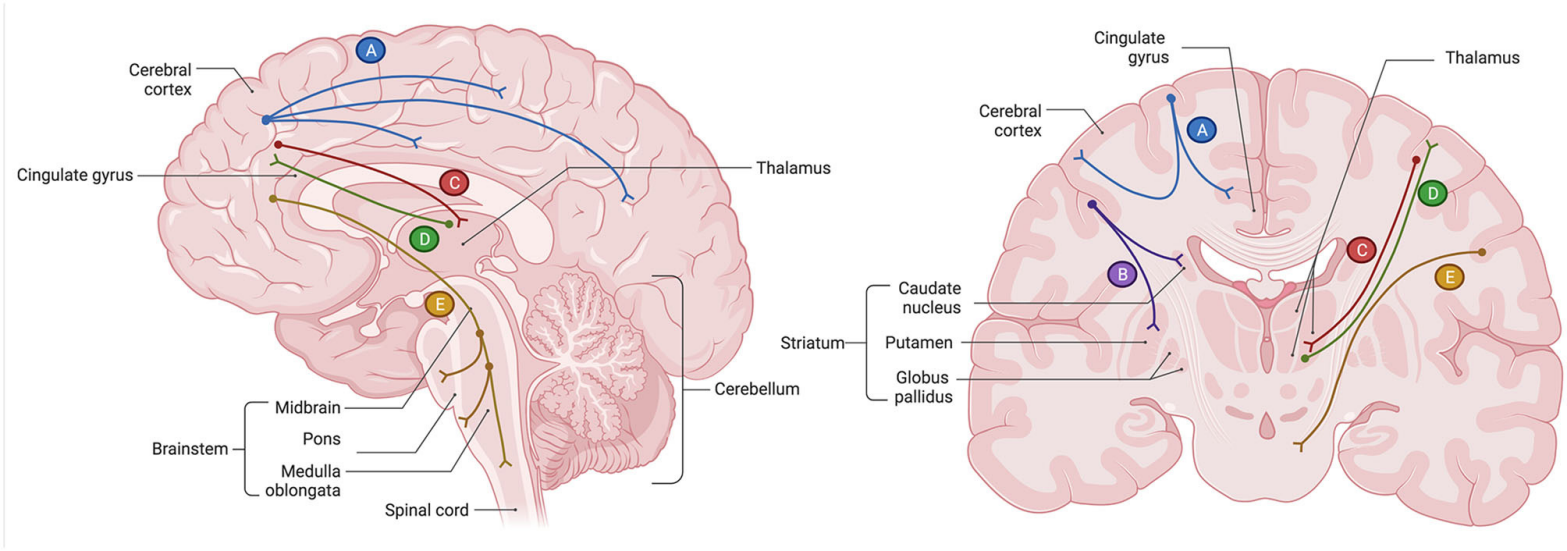
Glutamatergic circuitry of the human brain: five cortical pathways (35, 36). **(A)** The cortico-cortical glutamate pathways constitute regions within the cerebral cortex, including the prefrontal cortex (PFC), that communicate with each other *via* glutamatergic pyramidal neurons. Axons of those neurons form white matter commissural fibers that interconnect cortical regions between two cerebral hemispheres, as well as short (U-fibers) and long association fibers that interconnect cortical regions within the same cerebral hemisphere. **(B)** The cortico-striatal glutamate pathway is a descending pathway that originates in the PFC and projects to the regions of the striatum, including the nucleus accumbens (NAc) of the ventral striatum. This pathway comprises the “cortico-striatal” portion of cortico-striatal-thalamic loops. **(C)** The cortico-brainstem glutamate pathways are comprised of white matter projection fibers that originate in the pyramidal neurons of the cerebral cortex, including the PFC, and descend to the major neurotransmitter centers situated in the nuclei of the brainstem. This pathway regulates the release of other neurotransmitters, notably the monoamines norepinephrine, serotonin, and dopamine, as well as gamma-aminobutyric acid (GABA). **(D)** The cortico-thalamic glutamate pathway is a descending projection that originates in the pyramidal neurons of the cerebral cortex, including those of the PFC, and innervates the thalamus (TH). **(E)** The thalamo-cortical glutamate pathway is an ascending pathway that originates in the neurons of the TH and terminates in the pyramidal neurons of the cerebral cortex, including those of the PFC. All cortical glutamatergic pathways involve the PFC, which carries a functional significance to the cognitive, behavioral, and affective symptoms of major depressive disorder (MDD) when glutamatergic circuits are dysregulated. Created with BioRender.com, RRID:SCR_018361.

**Figure 2:**
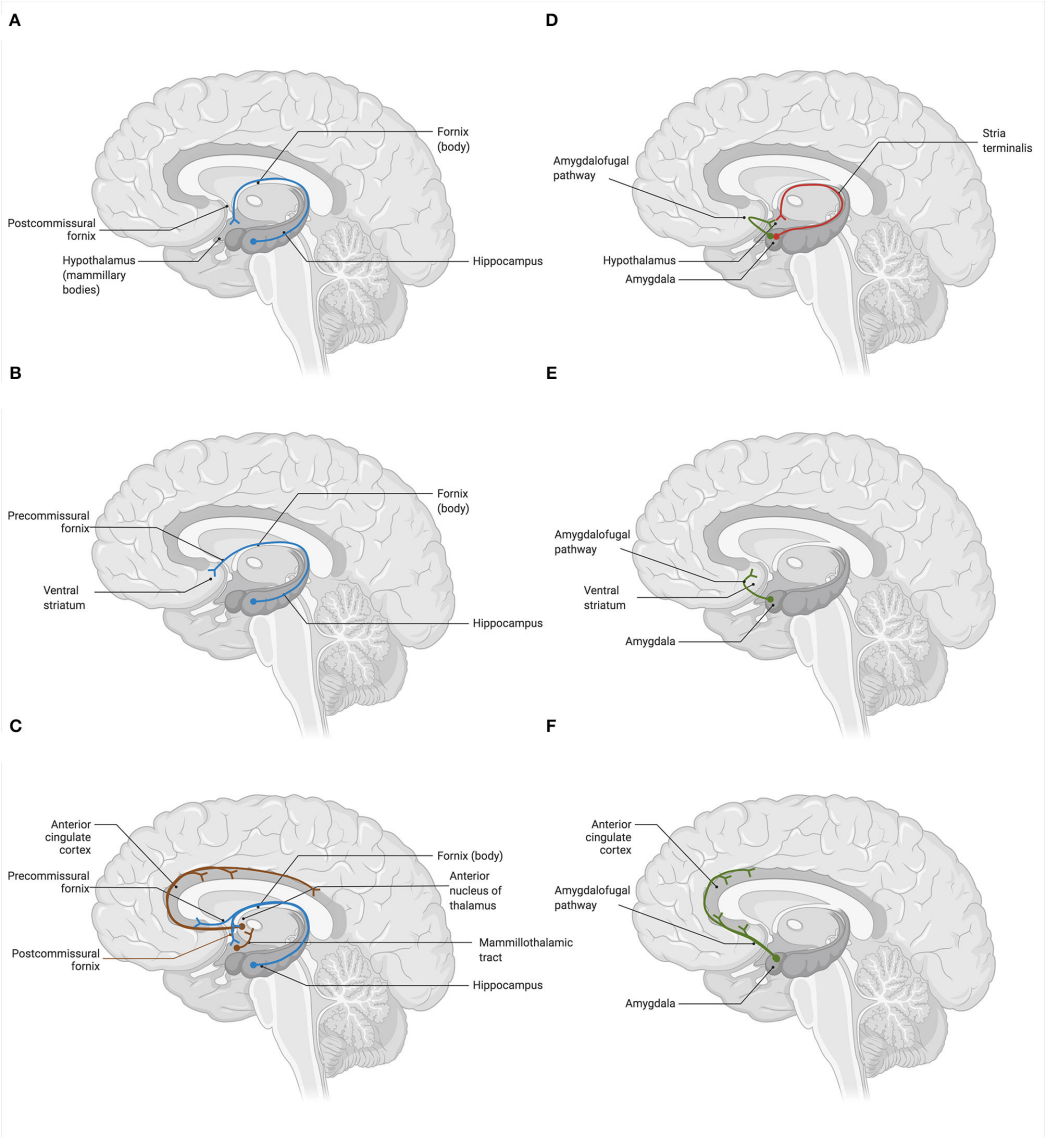
Glutamatergic circuitry of the human brain: hippocampal and amygdalar efferent pathways (37). Glutamatergic neurons deeply innervate the subcorticolimbic areas of the brain, comprising an integral part of the basic reward, affective, and memory circuits. Hippocampus (HPC) and amygdala (AMYG) are the key centers that are potentially affected in depression both structurally (i.e., gray matter volume, cell atrophy, dendritic, and axonal density) and functionally (i.e., cell physiology). **(A)** The hippocampal-hypothalamic glutamate pathway connects the HPC with the mammillary bodies of the hypothalamus (HPT) *via* the postcommissural branch of the fornix. **(B)** The hippocampal-striatal-tegmental glutamate pathway interconnects the HPC and the ventral striatum *via* the precommissural branch of the fornix. Neurons of the NAc subsequently project to the VTA, where they modulate the firing of dopaminergic neurons. **(C)** The hippocampal-cingulate glutamate pathways represent two pathways that interconnect the HPC and the anterior cingulate cortex (ACC). Neurons of the HPC innervate ACC directly, *via* the precommisural branch of the fornix and the entorhinal cortex, as well as indirectly, by passing through the anterior nucleus of the thalamus (TH) *via* the postcommissural branch of the fornix. In turn, the ACC projects back to the entorhinal cortex of the parahippocampal gyrus, forming the circuit of Papez. **(D)** The amygdalo-hypothalamic glutamate pathways include two major efferent pathways of the AMYG. These are the dorsal stria terminalis route, which connects the corticomedian nuclei of the AMYG to the lateral and ventral-medial nuclei of the HPT *via* the postcommissural branch of the stria terminalis, and the ventral amygdalofugal pathway, which connects the central and basolateral nuclei of the AMYG to the lateral HPT. **(E)** The amygdalo-striatal glutamate pathway projects from the central and basolateral nuclei of the AMYG to the areas of the ventral striatum, notably the NAc septi, *via* the ventral amygdalofugal pathway. **(F)** The amygdalo-cingulate glutamate pathway originates in the central and basolateral nuclei of the AMYG and innervates the ACC, including its dorsal and subgenual portions, *via* the ventral amygdalofugal pathway. Created with BioRender.com, RRID:SCR_018361.

In the human brain, several regions relevant to the core cognitive, behavioral, and affective functions are deeply interconnected *via* glutamatergic neurons. These include the prefrontal cortex (PFC), anterior cingulate cortex (ACC), nucleus accumbens (NAc), hippocampus (HPC), amygdala (AMYG), thalamus (TH), hypothalamus (HPT), and brainstem neurotransmitter centers regulating the release of norepinephrine (i.e., locus coeruleus), serotonin (i.e., raphe nuclei), dopamine [i.e., ventral tegmental area (VTA), substantia nigra pars compacta (SNc)], and GABA (i.e., substantia nigra pars reticulata). Broadly, brain glutamatergic networks can be categorized into cortical and subcorticolimbic. For the purposes of this review, we define cortical pathways as those with at least one node situated in the cerebral cortex, and subcorticolimbic pathways as those with no nodes in the cerebral cortex. The cortical glutamatergic pathways can be further subdivided into five major arcs (35, 36), illustrated in Figure 1. Similarly, the subcorticolimbic glutamatergic pathways can be categorized into hippocampal and amygdalar, where HPC and AMYG are the central limbic nodes (37), illustrated in Figure 2. Functionally, the subcorticolimbic pathways interconnect brain regions responsible for the generation of lower-order emotional and behavioral outputs in response to environmental inputs. Some notable examples include reward and motivation (NAc-VTA/SNc), memory formation and emotional engagement (HPC), detection of threat and activation of fear response (AMYG), and autonomic and homeostatic regulation (HPT). Cortical pathways, on the other hand, regulate lower-order outputs (“top-down” processing) and the subsequent fine-tuning of already existing higher-order representations (“bottom-up” processing). Taken together, due to the abundance of glutamatergic neurons, the transmission of glutamate through cortical and subcorticolimbic pathways accounts for the greatest proportion of the brain's functional connectivity profile, making it a principal mediator in the broad management and manifestation of complex cognitive and emotional processes.

## Glutamatergic Neurocircuitry Dysfunction in Major Depressive Disorder

A growing literature supports the notion that a widespread network dysconnectivity, as opposed to aberrant responses within select brain regions, is what characterizes and possibly drives pathophysiological changes associated with MDD (35, 38). Behind these changes, a variety of molecular processes related to glutamate cycling and release may be implicated, including the decreased expression NMDA-Rs (39–41), AMPA-Rs (41, 42), or mGluRs (43), which have been reported both in humans and animal models of depression, as well as in postmortem brains of suicide victims (39, 44). Ultimately, impaired glutamate neurotransmission would compromise synaptic connectivity, leading to reduced synaptogenesis and impaired cell signaling (45). However, these impairments are most likely non-generalized but rather involve structural and functional aberrations within specific nodes of major networks, for example, the PFC, HPC, or AMYG. Due to the highly interconnected nature of the brain, these regional alterations in node synaptic properties would likely further drive downstream changes in more distant nodes, causing widespread dysconnectivity of glutamatergic neurocircuitry both cortically and subcortically. Nevertheless, a definitive integrative pathway-focused framework that would describe how molecular changes in glutamate transmission contribute to brain connectivity impairments is yet to be proposed and experimentally validated.

However, in a similar fashion to how the monoamine deficiency hypothesis was proposed, the glutamate hypothesis initially attempted to associate the pathophysiology of depression with directional changes in absolute levels of glutamate within the synapse. For instance, existing *in vivo* proton magnetic resonance spectroscopy (^1^H-MRS) literature largely supports the hypoglutamatergic view of depression, supplemented with the robust evidence of the reduction in glutamate and GLX (glutamate + glutamine) levels in those with MDD (46). A review of 16 ^1^H-MRS studies, with a total of 281 MDD patients and 301 healthy controls (HC), reported significantly lower levels of glutamate and GLX in MDD participants, primarily in the ACC (47). Subsequently, the hypoglutamatergic hypothesis has been confirmed by a more recent meta-analysis on 1,180 MDD patients and 1,066 HC, which concluded that lower levels of GLX, primarily in the medial PFC (mPFC), were inherently linked with the etiology of MDD (48). The reports of successful restoration of GLX levels to normal that were observed in the ACC (49) and dorsolateral PFC (DLPFC) (50) of MDD patients post-electroconvulsive therapy (ECT) further support this notion. Several ^1^H-MRS studies, on the other hand, have reported increased (51–53) and unchanged (54, 55) glutamate/GLX levels in MDD participants, suggesting that the direction and magnitude of glutamate-specific alterations may differ depending on the brain region or the network of interest (56). Therefore, studying the whole-brain connectome as if comprised of a set of major ICNs, each having their corresponding functional and behavioral significance, may provide a more specific direction toward characterizing regional changes in absolute glutamate levels and how those could translate into specific symptoms of depression.

## Introduction to Intrinsic Connectivity Networks in Major Depressive Disorder

**Figure 3:**
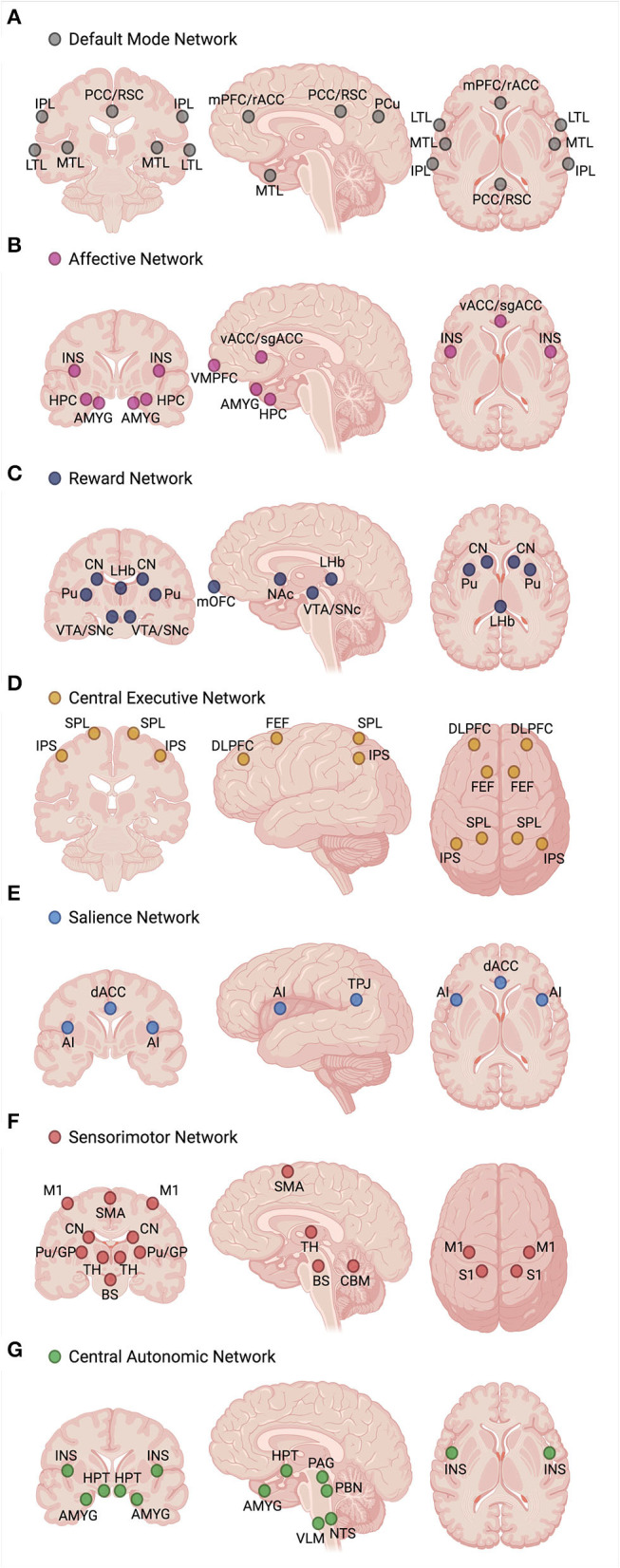
Functional profile of seven intrinsic connectivity networks (ICNs), including the **(A)** default mode network (DMN), **(B)** ventromedial affective network (AN), **(C)** ventral frontostriatal reward network (RN), **(D)** frontoparietal central executive network (CEN), **(E)** anterior cinguloinsular salience network (SN), **(F)** frontocerebellar sensorimotor network (SMN), and **(G)** frontovagal central autonomic network (CAN), has been associated with the pathophysiology and symptom manifestation in depression. Hyperconnectivity of the DMN, AN, and CAN and hypoconnectivity of the RN, CEN, SN, and SMN are the hallmark biomarker features that can differentiate depressed individuals from healthy controls. Disturbances in specific glutamatergic white matter pathways may form a neural substrate for aberrant functional connectivity within and between the core ICNs, serving as neuroanatomical targets for future mechanistic studies involving glutamate-based therapies. Created with BioRender.com, RRID:SCR_018361. AI, anterior insula; AMYG, amygdala; BS, brainstem (sensorimotor nuclei); CBM, cerebellum; CN, caudate nucleus; dACC, dorsal anterior cingulate cortex; DLPFC, dorsolateral prefrontal cortex; FEF, frontal eye fields; GP, globus pallidus; HPC, hippocampus; HPT, hypothalamus; INS, insula; IPL, inferior parietal lobule; IPS, intraparietal sulcus; LHb, lateral habenula; LTL, lateral temporal lobe; M1, primary motor cortex; mOFC, medial orbitofrontal cortex; mPFC, medial prefrontal cortex; MTL, medial temporal lobe; NAc, nucleus accumbens; NTS, nucleus tractus solitarius; PAG, periaqueductal gray; PBN, parabrachial nucleus; PCC, posterior cingulate cortex; PCu, precuneus; Pu, putamen; rACC, rostral anterior cingulate cortex; RSC, retrosplenial cortex; S1, primary somatosensory cortex; sgACC, subgenual anterior cingulate cortex; SMA, supplementary motor area; SNc, substantia nigra pars compacta; SPL, superior parietal lobule; TH, thalamus; TPJ, temporoparietal junction; vACC, ventral anterior cingulate cortex; VLM, ventrolateral medulla; VMPFC, ventromedial prefrontal cortex; VTA, ventral tegmental area.

While the exact number of ICNs and the functional role of each are not yet fully known, the consensus of neuroimaging studies has revealed the existence of 7–17 distinct functional ICNs based on stable network parcellations (57, 58). Multi-modal neuroimaging literature, including studies of functional magnetic resonance imaging (fMRI), electroencephalography (EEG), magnetoencephalography (MEG), and positron emission tomography (PET), have identified several candidate ICNs that are functionally relevant to the symptomatology and pathophysiology of MDD. In this review, we focus on seven candidate ICNs that have been consistently replicated in MDD neuroimaging studies, illustrated in Figure 3: the default mode network (DMN), ventromedial affective network (AN), ventral frontostriatal reward network (RN), frontoparietal central executive network (CEN), anterior cinguloinsular salience network (SN), frontocerebellar sensorimotor network (SMN), and frontovagal central autonomic network (CAN) (23, 38, 59–61).

Although the definition and characterization of these ICNs primarily emerge from the functional brain imaging data, each of these networks has a structural connectivity substrate that overlaps with anatomically defined glutamatergic pathways, proposed here based on the core nodes forming each ICN. Glutamatergic circuits existing in the human brain may interconnect brain areas within a single ICN, determining within-network connectivity patterns (Table 2A), as well as brain areas distinctly involved in two or more ICNs, determining between-network connectivity patterns (Table 2B). For instance, the DMN, the best known and the most studied ICN, comprises a reproducible set of regions active during stimulus-free control conditions of task-based neuroimaging (“at rest”) that become deactivated during a loaded cognitive task (62, 63). While it is known that central DMN nodes are mainly localized within the cerebral cortex, given the abundance of glutamatergic neurons in the brain (i.e., ~80%) and our understanding of neuroanatomy, cortical-cortical glutamatergic connections would constitute the majority of synapses within the DMN (edges), thus forming a substrate for its within- and between-network functional connectivity patterns. A similar theoretical approach could be applied to other ICNs, where our knowledge of the core network nodes and existing white matter tracts might shed light on the specific glutamatergic connections that possibly represent the edges of a network or a subnetwork (Tables 2A,B). While these assumptions are hypothetical and should be interpreted as such, they nevertheless rely on the well-described patterns of human white matter neuroanatomy and could greatly inform the neuroimaging community as to which particular glutamatergic connections within the brain may warrant attention of future investigations.

**Table 2A:** Anatomical and functional profile of glutamatergic intrinsic connectivity networks in depression.

**Intrinsic connectivity network**	**Key nodes**	**Glutamatergic edges (within-network)**	**Functions in depression**	**Connectivity in depression (within-network)**
Default mode network (DMN)	mPFC/rACC PCC/RSC, PCu, IPL MTL/LTL	Cortico-cortical Hippocampal-cingulate	Excessive self-referential processes, maladaptive rumination	↑
Affective network (AN)	vACC/sgACC, VMPFC AMYG, HPC, INS	Cortico-cortical Hippocampal-cingulate Amygdalo-cingulate	Negative affect, sensitivity to negative information, dysphoria	↑
Reward network (RN)	mOFC/VMPFC BG (CN, Pu, NAc, VTA/SNc) LHb	Cortico-striatal	Loss of pleasure, interest, motivation (anhedonia), inability to sustain positive affect	↓
Central executive network (CEN)	DLPFC, FEF SPL, IPS	Cortico-cortical	Disrupted cognitive and executive control, impaired top-down regulation of the limbic system	↓
Salience network (SN)	dACC AI TPJ	Cortico-cortical	Poor incentive salience, anhedonia, reduced goal-directed behavior, response selection/inhibition impairment	↓
Sensorimotor Network (SMN)	M1, SMA, S1 BG (CN, Pu, GP, STN, SNc) TH BS, CBM	Cortico-cortical Cortico-striatal Cotrico-thalamic Thalamo-cortical Cortico-brainstem	Psychomotor retardation, altered bodily awareness and pain perception	↓
Central autonomic network (CAN)	HPT PAG, PBN, NTS, VLM AMYG, INS	Cortico-brainstem Cortico-thalamic Thalamo-cortical Hippocampal-hypothalamic Amygdalo-hypothalamic	Increased responsivity to stress, arousal, changes in cardiovascular and respiratory functions, changes in the sleep cycle, biological rhythms, libido, and appetite	↑

*AI, anterior insula; AMYG, amygdala; BG, basal ganglia; BS, brainstem (sensorimotor nuclei); CBM, cerebellum; CN, caudate nucleus; dACC, dorsal anterior cingulate cortex; DLPFC, dorsolateral prefrontal cortex; FEF, frontal eye fields; GP, globus pallidus; HPC, hippocampus; HPT, hypothalamus; INS, insula; IPL, inferior parietal lobule; IPS, intraparietal sulcus; LHb, lateral habenula; LTL, lateral temporal lobe; M1, primary motor cortex; mOFC, medial orbitofrontal cortex; mPFC, medial prefrontal cortex; MTL, medial temporal lobe; NAc, nucleus accumbens; NTS, nucleus tractus solitarius; PAG, periaqueductal gray; PBN, parabrachial nucleus; PCC, posterior cingulate cortex; PCu, precuneus; Pu, putamen; rACC, rostral anterior cingulate cortex; RSC, retrosplenial cortex; S1, primary somatosensory cortex; sgACC, subgenual anterior cingulate cortex; SMA, supplementary motor area; SNc, substantia nigra pars compacta; SPL, superior parietal lobule; STN, subthalamic nucleus; TH, thalamus; TPJ, temporoparietal junction; vACC, ventral anterior cingulate cortex; VLM, ventrolateral medulla; VMPFC, ventromedial prefrontal cortex; VTA, ventral tegmental area. ↑, increased; ↓, decreased*.

**Table 2B:** Proposed anatomical white matter substrate for glutamate-mediated within- and between-network connectivity.

**Connectivity**	**DMN**	**AN**	**RN**	**CEN**	**SN**	**SMN**	**CAN**
DMN	• *Cortico-cortical* • *Hippocampal-cingulate*	• Cortico-cortical • Hippocampal-cingulate • Amygdalo-cingulate	• Cortico-cortical • Cortico-striatal • Hippocampal-striatal-tegmental	• Cortico-cortical	• Cortico-cortical • Hippocampal-cingulate	• Cortico-cortical • Cortico-striatal • Cortico-brainstem • Cortico-thalamic • Thalamo-cortical • Hippocampal-striatal-tegmental	• Cortico-brainstem • Cortico-thalamic • Thalamo-cortical • Hippocampal-hypothalamic • Amygdalo-cingulate
AN	• Cortico-cortical • Hippocampal-cingulate • Amygdalo-cingulate	• *Cortico-cortical* • *Hippocampal-cingulate* • *Amygdalo-cingulate*	• Cortico-cortical • Cortico-striatal • Hippocampal-striatal-tegmental • Amygdalo-striatal	• Cortico-cortical	• Cortico-cortical • Hippocampal-cingulate • Amygdalo-cingulate	• Cortico-cortical • Cortico-striatal • Cortico-brainstem • Cortico-thalamic • Thalamo-cortical • Hippocampal-striatal-tegmental • Amygdalo-striatal	• Cortico-brainstem • Cortico-thalamic • Thalamo-cortical • Hippocampal-hypothalamic • Amygdalo-hypothalamic
RN	• Cortico-cortical • Cortico-striatal • Hippocampal-striatal-tegmental	• Cortico-cortical • Cortico-striatal • Hippocampal-striatal-tegmental • Amygdalo-striatal	• *Cortico-striatal*	• Cortico-cortical • Cortico-striatal	• Cortico-cortical • Cortico-striatal	• Cortico-cortical • Cortico-striatal • Cortico-brainstem • Cortico-thalamic • Thalamo-cortical	• Cortico-brainstem • Cortico-thalamic • Thalamo-cortical • Amygdalo-striatal
CEN	• Cortico-cortical	• Cortico-cortical	• Cortico-cortical • Cortico-striatal	• *Cortico-cortical*	• Cortico-cortical	• Cortico-cortical • Cortico-striatal • Cortico-brainstem • Cortico-thalamic • Thalamo-cortical	• Cortico-brainstem • Cortico-thalamic • Thalamo-cortical
SN	• Cortico-cortical • Hippocampal-cingulate	• Cortico-cortical • Hippocampal-cingulate • Amygdalo-cingulate	• Cortico-cortical • Cortico-striatal	• Cortico-cortical	• *Cortico-cortical*	• Cortico-cortical • Cortico-striatal • Cortico-brainstem • Cortico-thalamic • Thalamo-cortical	• Cortico-brainstem • Cortico-thalamic • Thalamo-cortical
SMN	• Cortico-cortical • Cortico-striatal • Cortico-brainstem • Cortico-thalamic • Thalamo-cortical • Hippocampal-striatal-tegmental	• Cortico-cortical • Cortico-striatal • Cortico-brainstem • Cortico-thalamic • Thalamo-cortical • Hippocampal-striatal-tegmental • Amygdalo-striatal	• Cortico-cortical • Cortico-striatal • Cortico-brainstem • Cortico-thalamic • Thalamo-cortical	• Cortico-cortical • Cortico-striatal • Cortico-brainstem • Cortico-thalamic • Thalamo-cortical	• Cortico-cortical • Cortico-striatal • Cortico-brainstem • Cortico-thalamic • Thalamo-cortical	• *Cortico-cortical* • *Cortico-striatal* • *Cortico-brainstem* • *Cotrico-thalamic* • *Thalamo-cortical*	• Cortico-brainstem • Cortico-thalamic • Thalamo-cortical • Amygdalo-striatal • Amygdalo-cingulate
CAN	• Cortico-brainstem • Cortico-thalamic • Thalamo-cortical • Hippocampal-hypothalamic • Amygdalo-cingulate	• Cortico-brainstem • Cortico-thalamic • Thalamo-cortical • Hippocampal-hypothalamic • Amygdalo-hypothalamic	• Cortico-brainstem • Cortico-thalamic • Thalamo-cortical • Amygdalo-striatal	• Cortico-brainstem • Cortico-thalamic • Thalamo-cortical	• Cortico-brainstem • Cortico-thalamic • Thalamo-cortical • Amygdalo-cingulate	• Cortico-brainstem • Cortico-thalamic • Thalamo-cortical • Amygdalo-striatal	• *Cortico-brainstem* • *Cortico-thalamic* • *Thalamo-cortical* • *Hippocampal-hypothalamic* • *Amygdalo-hypothalamic*

*Intrinsic Connectivity Networks: AN, ventromedial affective network; CAN, frontovagal central autonomic network; CEN, frontoparietal central executive network; DMN, default mode network; RN, ventral frontostriatal reward network; SMN, frontocerebellar sensorimotor network; SN, anterior cinguloinsular salience network. Within-network connectivity pathways are italicized.*

When considering cognitive impairment and prominent affective dysregulation characterizing MDD, a network model of depression views these deficits, as well as other MDD symptoms, as the manifestation of altered connectivity within and between major ICNs (Figure 3) (38). To summarize, enhanced DMN connectivity is associated with excessive self-referential processes and maladaptive rumination (64–67). Similarly, elevated connectivity of the AN, a crucial network linked with the processing and regulation of emotions, may underlie excessive negative feelings, or dysphoria (68–70). Conversely, attenuated RN connectivity has been suggested to account for the symptoms of anhedonia, characterized by the loss of pleasure, reward, and motivation, as well as reduced magnitude and duration of positive affect upon exposure to positive stimuli (71–74). The SN, responsible for task-switching and goal-directed behaviors, also appears to be hypoactive in MDD, which translates into poor incentive salience, impaired response inhibition and selection, and diminished ability to initiate a behavior through premotor and motor regions, thus further contributing to anhedonia (64, 75–77). In turn, depression is also characterized by cognitive and executive deficits, marked by inefficient top-down regulation of emotions and negative thinking. Impaired top-down control is thought to be attributed to diminished CEN connectivity, which has been seen in depressed individuals at rest (64, 78–80) and during tasks involving working memory (81), executive control (82), and affective interference (83). Hypoconnectivity within the SMN has been linked with the symptoms of psychomotor retardation (84, 85), and several studies have shown this association among patients with MDD (30, 86–88). A relatively “new” ICN incorporated into the network model is the CAN, which involves the HPT, AMYG, insula, and brainstem nuclei responsible for the control of bodily autonomic responses (61, 89). The HPT appears to be the central node of the CAN that integrates autonomic, endocrine, and sleep functions, and CAN hyperconnectivity possibly accounts for the strong association between depression and physiological mechanisms inappropriately regulated by the autonomic nervous system, including the distinct markers of elevated heart rate, low heart rate variability, increased arousal, and responsivity to stress. All these subsequently translate into changes in the sleep cycle, neuroendocrine response, sexual function, and appetite that are frequently observed in MDD patients.

While within-network connectivity within each of the seven core ICNs could potentially represent a neural substrate for specific affective, cognitive, behavioral, and physiological constructs of depression, the interaction among their respective nodes is far more complex. Functional communication between the ICNs, or between-network connectivity, also plausibly contributes to the expression of depressive phenotypes. For instance, the state of negative self-referential thinking has been attributed to disrupted between-network connectivity of the DMN and AN with the SN and CEN, as well as the hyperactive subgenual anterior cingulate cortex (sgACC)—a central node of the AN (23, 90). Specifically, the posterior DMN displays elevated connectivity with the SN and CEN, while the anterior DMN displays diminished connectivity with the AN and SN. The connectivity between the SN and AN also appears to be reduced in depression. Hence, it is not the elevated connectivity within the DMN alone but also the interaction between the specific DMN nodes and nodes of other ICNs that account for the overall manifestation of the functional profile of negative self-referential thinking. Therefore, when considering ICNs as biomarkers and biologically plausible therapeutic targets, one should not treat ICNs as distinct units but should view them as integrated whole-brain functional circuits. In fact, the basal ganglia (91) and cerebellum (92) have their own ICNs forming their respective organization and topography, which further supports this notion.

## Glutamate and Intrinsic Connectivity Networks in Major Depressive Disorder

Since ICNs, at large, constitute functional rather than structural units, determining the neurochemical source driving ICN changes is challenging. ^1^H-MRS is a specialized neuroimaging technique that enables *in vivo* quantification of metabolites within pre-specified regions of interest, and among them, the concentrations of glutamate, glutamine, and GLX (93). Although this technique is subject to confounds, such as functional status of recruited samples, magnet strength (3T vs. 7T), and exposure to medication (94), pairing ^1^H-MRS with functional neuroimaging modalities may partially mitigate the challenge of determining the neurochemical source of signal, providing an insight into the role of glutamate in the circuits involved in the pathophysiology of depression (95). While the interpretation of neuroimaging findings is generally constrained by the inverse problem, where the source of observed signals can be inferred only indirectly, the abundance of glutamatergic neurons (~80%) (33) and the role of glutamate in neuroenergetics (96–98) may in part account for ICN-related changes.

Mechanistically, glutamatergic signaling contributes to the excitation-inhibition balance responsible for the generation of neural oscillations that underlie slow fluctuations of neuronal activity visualized by means of brain imaging (99, 100). In other words, glutamate is a neurotransmitter that orchestrates synchronized neuronal activity across the entire brain, and the temporal correlation between these patterns of activation is what comprises the ICNs. The emerging literature has actively examined the relationship between glutamate levels and functional network small-world and global properties. As demonstrated by *in vitro* experiments, acute glutamate treatment leads to increases in similarity and connectivity weight between cultured neuronal networks, implicating enriched communication between neurons and stronger functional connection (101, 102). Animal studies have demonstrated an association between glutamatergic neurotransmission, neuronal firing rate, and blood-oxygen-level-dependent (BOLD) signal in the rat brain (103, 104), while *in vivo* human multi-imaging studies have shown that glutamate plays a vital role in modulating BOLD response both at rest and during functional tasks (98, 105, 106), as well as the functional connectivity between brain regions (105, 107–114). Additionally, a recent systematic review and meta-analysis of ^1^H-MRS-fMRI studies combining the two modalities reported converging evidence supporting a positive association between glutamate levels and local (activity inside the spectroscopy voxel) as well as distal (activity outside the spectroscopy voxel) task-related brain activity (115). This evidence indicates that glutamate exerts a global effect on the BOLD response *via* abundant long-range glutamatergic projections to other cortical areas rather than modulating a local BOLD response within the acquired ^1^H-MRS voxel (111, 116).

From this standpoint, changes in synchronized neuronal oscillations caused by disrupted glutamate neurotransmission may indeed be associated with the aberrant dynamics of ICNs, and the relationship between glutamate levels and ICN activity and connectivity has been confirmed in psychiatric populations. ^1^H-MRS studies have demonstrated an association between altered metrics corresponding to glutamate levels and functional changes in the nodes of the DMN (108, 109, 112, 117), AN (118–123), RN (124, 125), CEN (126, 127), SN (98, 110, 116, 128), SMN (129), and CAN (130). Of note, a study by Levar et al. (126), which examined the relationship between glutamate levels in the dorsal ACC (dACC) (i.e., the core SN node), as well as the within-network connectivity of the DMN, SN, and CEN, reported no significant association between resting-state connectivity patterns in any of the ICNs and absolute glutamate levels (126). However, low GABA/glutamate ratios were linked with elevated connectivity within the DMN and SN, suggesting that the interplay between GABA and glutamate levels, rather than absolute glutamate levels, might drive network functional connectivity.

Given the plausibility that functional connectivity of ICNs might be positively correlated with absolute or relative glutamate levels between the synapses, the brain network model fails to support the absolute state of cortical hypo- or hyperglutamatergia in depression. Instead, in an MDD patient, glutamate levels would be dynamically altered as a function of ICN involvement during a particular mental state or task engagement, representing a “functional pathology” as opposed to a static neurochemical one. For instance, glutamate levels might remain inappropriately elevated at rest during the DMN involvement but would be pathologically diminished when the engagement of the SN or CEN is warranted (i.e., switching from the resting state to the state of information processing). Furthermore, another hypothetical assumption is that baseline glutamate levels in depression may vary across the cortex based on the neuroanatomy of specific ICN nodes and edges. For instance, cortico-cortical tracts between mPFC/rostral ACC (rACC) and posterior cingulate cortex (PCC) (i.e., the core DMN nodes) might have increased glutamatergic neurotransmission, while cortico-cortical tracts interconnecting dACC and anterior insula (AI) (i.e., the core SN nodes) might display diminished glutamatergic neurotransmission. This is despite the fact that both tracts pass in anatomical proximity to each other through the cingulum bundle, given that the nodes of DMN and SN are localized within rACC and dACC, respectively. From the mechanistic point of view, targeting the networks selectively makes treatment particularly challenging since currently available glutamatergic compounds lack this specificity and precision, and their basic pharmacodynamic properties do not get modulated by specific mental states.

In the following section of the article, we review the present state of evidence from studies that utilized glutamatergic treatments to investigate changes in ICN functional dynamics in MDD participants. The ICN changes discussed in this review emerge from studies that administered glutamate-modulating compounds as treatment for MDD/TRD, and most studies did not directly measure glutamate, glutamine, or GLX levels (Table 3). Therefore, these findings should be interpreted with caution, as the association between ICNs and glutamate signaling is presumed based on understanding the pharmacodynamics of administered compounds and not the direct association between neuroimaging and neurochemical metrics. We present the results of the reviewed studies grouped into the effects of glutamatergic interventions on ICN functional metrics (Table 4A) and the ICN biomarkers that predict antidepressant response (Table 4B). Observed changes in specific ICNs would provide mechanistic insights into the etiology of brain network abnormalities in depression. This is the first step toward designing prospective glutamate-focused ^1^H-MRS-fMRI whole-brain connectivity studies, furthering the development of future glutamate-mediating treatments to selectively target these functional abnormalities within the brain connectome.

**Table 3:** Neuroimaging methods of reviewed studies.

**Glutamatergic compound**	**References**	**Imaging sample size (MDD, HC)**	**Imaging technique**	**ROIs for activity/cerebral blood flow/entropy/receptor binding/glucose metabolism measurement**	**Methods for connectivity measurement**	**Timepoint(s) of MDD imaging relative to treatment**
Ketamine	Abdallah et al. (131)	18, 25	fMRI	N/A	Average correlation between BOLD time series of a voxel and all other gray matter voxels in brain (GBCr)	Pre-treatment and 24 h post-treatment
	Abdallah et al. (132)	22 (Cohort A only), 29 HC in Cohort A and 18 HC in Cohort B	fMRI	N/A	Average correlation between BOLD time series of a voxel and all other gray matter voxels in brain (GBCr)	• Cohort A: pre-treatment and 24 h post-treatment • Cohort B: pre-treatment and repeated during each treatment session
	Abdallah et al. (133)	56, 0	fMRI	N/A	Average correlation between BOLD time series of a voxel and all other gray matter voxels in brain (GBCr)	Pre-treatment, during infusion, and 24 h post-treatment
	Carlson et al. (134)	20, 0	^18^F-FDG-PET	• Whole-brain CMRGlu • ROIs in AMYG, sgACC, anterior HPC, and medial TH	N/A	Pre-treatment and ~120 min post-treatment
	Chen et al. (135)	48, 0	fMRI	N/A	• Six ROIs in prefrontal regions (dACC, DLPFC, and mPFC) • Seeds defined as structures with a 4 mm radius around the coordinates • FC map of ROIs identified by correlating low-frequency fMRI fluctuations with the seeds	Pre-treatment and on third day after treatment (~48 h post-treatment)
	Chen et al. (136)	48, 48	fMRI	N/A	• Four striatal seeds (limbic, executive, rostral-motor, caudal motor) • FC map of striatum identified by correlating low-frequency fMRI fluctuations with the ROIs	Pre-treatment
	Downey et al. (137)	56, 0	fMRI	ROI in sgACC (x = 0, y = 26, z = −8 mm)	N/A	Pre-treatment and 1 h scanning session which treatment was administered during
	Evans et al. (138)	33, 25	fMRI	N/A	• DMN defined using seed-based correlation method (3dTcorr) • Average time course from 6 mm radius sphere at PCC (3dROIstats) at the MNI template coordinates of (0, 252, 27) correlated with all other brain voxels	Pre-treatment and ~2 and 10 days after both infusions
	Gärtner et al. (139)	24, 0	fMRI	N/A	• ROIs (MNI): sgACC (sphere at 2, 28, −5 with 5 mm radius), the left and right DLPFC (sphere at ±40, 36, 32 with 10 mm radius), the left and right PCC (sphere at ±6, −50, 24 with 7 mm radius), and the left and right HPC (anatomical seeds from the FSL Harvard-Oxford Atlas) • Seed-to-voxel correlation maps calculated by extracting residual BOLD time course from seed and computing correlation between that time course and time course of all other voxels; GLM	Pre-treatment and 24 h post-treatment
	Gilbert et al. (140)	29, 0	MEG	ROIs in left AI (Talairach coordinates: −32, 13, −8) and ACC (Talairach coordinates: −2, 43, 2)	N/A	Pre-treatment and 6–9 h post-treatment
	Kraus et al. (141)	28, 22	fMRI	N/A	• GBC map transformed to MNI; maps resampled to final voxel size of 3.5 mm isotropic (voxel volume: 42.875 mm^3^) • Intra-PFC GBC calculated using same procedures, but GBC calculations restricted to the PFC with a PFC GM mask	Pre-treatment and day 2 or 3 after ketamine or placebo
	Lally et al. (142)	20, 0	^18^F-FDG-PET	Whole-brain CMRGlu and ROIs in ventral striatum and OFC	N/A	Pre-treatment and 2 h post-treatment
	Li et al. (143)	48, 0	^18^F-FDG-PET	• ROIs in AMYG and PFC • Whole-brain voxel-wise analyses	N/A	Pre-treatment and immediately after treatment
	Loureiro et al. (144)	44, 31	fMRI	Whole-brain analysis and ROI in AMYG	N/A	Pre-treatment and 24–72 h after last infusion
	Loureiro et al. (145)	46, 32	fMRI	• Cluster in CBM in lobule-VIIb in dorsal-attention part of CBM defined as psychophysiological-interaction seed • ROIs for CEN (lateral OFC, inferior frontal cortex, supramarginal gyrus, and DLPFC), SN (AI, dACC, and middle frontal cortex) and SMN (posterior INS, precentral and the superior paracentral)	Psychophysiological-interaction analysis using CBM seed generated from the average NoGo-Go activation; GLM	Pre-treatment, 24 h after first infusion, and 24 or 72 h after last infusion
	McMillan et al. (146)	26, 0	fMRI and EEG	sgACC	N/A	Treatment administered 7 min into a 16 min scan
	Mkrtchian et al. (147)	27, 19	fMRI	N/A	• Seed-to-whole-brain analysis with four striatal seeds (3.5 mm radius spheres): ventral striatum (±9, 9, −8), dorsal caudate (±13, 15, 9), dorsal caudal Pu (±28, 1, 3), and ventral rostral Pu (±20, 12, −3) • Primary visual cortex used as control region for sensitivity analysis examining whether results were specific to PFC regions or due to global pattern	2 days post-treatment
	Morris et al. (148)	42, 20	fMRI	ROI in sgACC	Computed between sgACC and HPC ROI's using Pearson's correlation	Pre-treatment and within 5 days after infusion
	Murrough et al. (149)	18, 20	fMRI	• Whole-brain, voxel-wise GLM • Single-subject whole-brain maps reflecting BOLD signals	Investigated FC of regions that demonstrated brain activation main effects; GLM	Pre-treatment and 24 h post-treatment
	Nakamura et al. (150)	15, 0	fMRI	N/A	• Whole brain FC maps with seed regions for each hemispheric AMYG and bilateral ventral PCu in the MNI space • FC between seed and each voxel of whole brain computed as contrast of parameter estimates of the GLM	Pre-treatment and 6–24 h after last infusion
	Nemati et al. (151)	258 total (randomized participants were scanned), 0	fMRI	N/A	GBC computed with average correlation of each voxel/vertex with all other gray matter voxels and vertices	Pre-treatment and during infusion
	Nugent et al. (152)	13, 18	MEG and ^18^F-FDG-PET	N/A	• Data from subjects entered into group independent components analysis to extract 25 components; linear regression used to obtain independent component maps • ROIs in left and right AMYG and sgACC	Pre-treatment, MEG 6–7 h post-treatment, ^18^F-FDG-PET ~120 min post-treatment
	Reed et al. (153)	33, 26	fMRI	Whole brain analysis	N/A	Pre-treatment and 1–3 days after each infusion
	Reed et al. (154)	33, 24	fMRI	Whole brain analysis	N/A	Pre-treatment and 1–3 days after each infusion
	Roy et al. (155)	11, 0	fMRI	• 132 ROIs • Cortical and subcortical from the FSL Harvard-Oxford atlas • Cerebellar regions from the automated anatomical labeling atlas	N/A	Pre-treatment and 1 day after last infusion
	Sahib et al. (156)	22, 18	Perfusion MRI	Regional cerebral blood flow values from ROIs that showed significant changes in whole brain analysis	N/A	Pre-treatment, 24 h after first infusion, 24–72 h after last infusion
	Sahib et al. (157)	47, 32	fMRI	• Whole brain analysis • ROIs in right and left SMA	N/A	Pre-treatment, 24 h after first infusion, 24–72 h after last infusion
	Sahib et al. (158)	61, 40	fMRI	N/A	• Nodes generated with high-dimensional group independent components analysis • Network modeling performed using partial temporal correlation between node time series	Pre-treatment, 24 h after first infusion, 24–72 h after last infusion
	Salvadore et al. (159)	11, 11	MEG	• False discovery rate calculated using a ROI approach encompassing the ACC/Brodmann area 24/32 • AMYG exploratory analyses	N/A	Pre-treatment
	Salvadore et al. (160)	15, 0	MEG	• False discovery rates estimated within ROI encompassing the ACC/Brodmann area 24/32	• Dynamic imaging of coherent sources using single seed point in the pgACC • ROI encompassing bilateral AMYG	Pre-treatment
	Siegel et al. (161)	23, 27	fMRI	N/A	Exploratory FC analysis to visualize connectivity between three targets (DMN, sgACC, limbic system) and the rest of the brain	Pre-treatment and 2 weeks post-treatment
	Sterpenich et al. (162)	10, 0	fMRI	• Whole brain analysis with anatomical masks for AMYG, INS, ACC, OFC, and ventral striatum • ROIs in medial substantia nigra/VTA	N/A	Pre-treatment and 1 and 7 days post-treatment
	Thai et al. (163)	11, 0	fMRI	ROIs in left and right HPC, left and right AMYG, subcallosal cortex, ACC, left and right NAc, PCu, and PCC	N/A	Pre-treatment and 1 day following last infusion
	Tiger et al. (164)	30, 0	PET	ROI in CBM	N/A	Pre-treatment and 24–72 h post-treatment
	Vasavada et al. (165)	44, 50	fMRI	N/A	• DMN, CEN, and SN chosen to investigate FC with HPC (right and left) and AMYG (right and left) • Time courses for AMYG and HPC extracted ROI masks derived from the Harvard–Oxford subcortical structural atlases • Correlations calculated between time courses of the networks and seeds	Pre-treatment, 24 h after first infusion, 24–72 h after last infusion
	Wang et al. (166)	60 (MDD and bipolar without psychotic symptoms), 0	fMRI	N/A	• Left and right habenula identified with centers of MNI coordinates (−2.8, −24.2, 2.3) and (4.8, −24.1, 2.2), respectively • Both of which have a radius of 3 mm	Pre-treatment
Memantine	Kilpatrick et al. (167)	26, 0	fMRI	N/A	Anterior and posterior DMN nodes (PCu and mPFCs) with dual regression to create individual DMN parameter estimate maps	Pre-treatment and 3-month follow-up
D-cycloserine	Cole et al. (168)	10, 12	EMG	M1 localized using neuronavigation and electromyographic electrodes positioned over right first dorsal interosseous muscle	N/A	1–2 h post-treatment
Nitrous oxide	–	–	–	–	–	–
4-chlorokynurenine	Park et al. (169)	12, 0	^1^H-MRS and fMRI	pgACC	30 ROIs previously identified as being associated with MDD using a 6 mm sphere	Pre-treatment and ~60–120 min post-treatment
Lanicemine	Abdallah et al. (133)	56, 0	fMRI	N/A	Average correlation between BOLD time series of a voxel and all other gray matter voxels in brain (GBCr)	Pre-treatment, during infusion, and 24 h post-treatment
	Downey et al. (137)	56, 0	fMRI	ROI in sgACC (x = 0, y = 26, z = −8 mm)	N/A	Pre-treatment and 1 h scanning session which treatment was administered during

*^1^H-MRS, proton magnetic resonance spectroscopy; ^18^F-FDG, fluorodeoxyglucose; AI, anterior insula; ACC, anterior cingulate cortex; AMYG, amygdala; BOLD, blood-oxygen-level-dependent; CBM, cerebellum; CEN, central executive network; CMRGlu, cerebral metabolic rate for glucose utilization; CN, caudate nucleus; dACC, dorsal anterior cingulate cortex; DLPFC, dorsolateral prefrontal cortex; DMN, default mode network; EEG, electroencephalogram; EMG, electromyography; FC, functional connectivity; fMRI, functional magnetic resonance imaging; GBC, global brain connectivity; GBCr, global brain connectivity with global signal regression; GLM, general linear modeling; GM, gray matter; HC, healthy control subjects; HPC, hippocampus; INS, insula; MDD, major depressive disorder participants; MEG, magnetoencephalography; MNI, Montreal Neurological Institute; mPFC, medial prefrontal cortex; MRI, magnetic resonance imaging; N/A, not applicable/available; NAc, nucleus accumbens; OFC, orbitofrontal cortex; PCC, posterior cingulate cortex; PCu, precuneus; PET, positron emission tomography; PFC, prefrontal cortex; pgACC, pregenual anterior cingulate cortex; Pu, putamen, ROI, region of interest; sgACC, subgenual anterior cingulate cortex; SMA, supplementary motor area; SN, salience network; TH, thalamus; VTA, ventral tegmental area*.

**Table 4A:** Effect of glutamatergic interventions on intrinsic connectivity networks.

**Glutamatergic compound**	**References**	**DMN**	**AN**	**RN**	**CEN**	**SN**	**SMN**	**CAN**
Ketamine	Abdallah et al. (131)	*↓ mPFC/DMPFC ↔ DLPFC*	*↓ sgACC ↔ DLPFC*	–	↑ right DLPFC *↓ DLPFC ↔ sgACC* *↓ DLPFC ↔ mPFC/DMPFC*	–	↓ left CBM	–
	Abdallah et al. (132)	↑ bilateral mPFC/DMPFC	–	–	↑ right DLPFC	–	–	–
	Abdallah et al. (133)	↑ DMPFC	–	–	↑ DLPFC	–	–	–
	Carlson et al. (134)	↑left _ _ _IPL	↑right _ _ _ _ _AMYG _↓right _ _INS _ _↓bilateral _ _ _ _vACC _↓ left_ _ _ _ _AMYG	↓right _ _ _LHb	↓right _ _ _ _ _DLPFC	↓right _ _AI	↑right _ _S1	↑right _ _ _ _ _AMYG _↓right _ _INS _ _↓left _ _ _ _ _AMYG
	Chen et al. (135)	↑*right anterior LTL ↔ right dACC* *↓ left MTL ↔ right dACC* *↓ left mPFC ↔ right dACC* *↓ right mPFC/frontal pole ↔ right DLPFC*	*↓ right vACC/sgACC ↔ left dACC*	–	*↓ right DLPFC ↔ right mPFC/frontal pole* ↑*right DLPFC ↔ left SPL ↓ right SPL ↔ left dACC ↓ left SPL ↔ right dACC*	*↓ left dACC ↔ right vACC/sgACC* ↑*right dACC ↔ right anterior LTL* *↓ left dACC ↔ right SPL* *↓ right and left dACC ↔ left M1* *↓ right dACC ↔ left MTL* *↓ right dACC ↔ left mPFC* *↓ right dACC ↔ left SPL*	*↓ left M1 ↔ left and right dACC*	–
	Chen et al. (136)	–	–	–	–	–	–	–
	Downey et al. (137)	↑**rACC** ↑**right PCC** ↑**bilateral LTL**	↑**vACC/sgACC**	↑**bilateral CN**	–	↑**bilateral dACC**	↑**bilateral TH** ↑**bilateral CN** ↑**bilateral CBM** ↑**bilateral BS**	↑**bilateral BS**
	Evans et al. (138)	↑*PCC ↔ right INS* ↑*PCC ↔ M1* ↑*PCC ↔ S1* ↑*PCC ↔ TH*	↑*right INS ↔ PCC*	–	–	↑*right AI ↔ PCC*	↑*M1 ↔ PCC* ↑*S1 ↔ PCC* ↑*TH ↔ PCC*	↑*right INS ↔ PCC*
	Gärtner et al. (139)	–	↑*sgACC ↔ right DLPFC*	–	*↑ right DLPFC ↔ sgACC*	–	–	–
	Gilbert et al. (140)	–	–	–	–	–	–	–
	Kraus et al. (141)	–	–	–	–	–	–	–
	Lally et al. (142)	–	↑right _ _ _ _HPC ↓ mOFC	↓mOFC	–	↑dACC	–	–
	Li et al. (143)	–	↓parahippocampus	–	–	↑dACC	↑SMA ↑ TH	–
	Loureiro et al. (144)	–	↓**right and left AMYG** ↑ **INS**	–	↑**right DLPFC**	↑**AI**	–	↓**right and left AMYG** ↑ **INS**
	Loureiro et al. (145)	–	–	–	*↓ CEN ↔ CBM*	*↓ SN ↔ CBM*	*↓ CBM ↔ SMN* *↓ CBM ↔ CEN* *↓ CBM ↔ SN*	–
	McMillan et al. (146)	↓**mPFC** _ ↑ **LTL** _ ↑ **PCC** _↑ **PCu**	↓**sgACC** ↓**HPC** ↓**right AMYG** ↑**right INS**	↑**right CN**	–	↑**dACC**	↓**S1** ↓ **M1**	↑**right INS**
	Mkrtchian et al. (147)	–	↑*mOFC ↔ ventral rostral Pu*	*↑ dorsal CN ↔ right ventrolateral PFC* *↑ dorsal caudal Pu ↔ pgACC* ↑*ventral striatum ↔ left DLPFC* ↑*mOFC ↔ ventral rostral Pu*	↑*left DLPFC ↔ ventral striatum*	–	↑*ventral rostral Pu ↔ mOFC* *↑ dorsal CN ↔ right ventrolateral PFC* *↑ dorsal caudal Pu ↔ pgACC* ↑*ventral striatum ↔ left DLPFC*	–
	Morris et al. (148)	–	↓**sgACC**	–	–	–	–	–
	Murrough et al. (149)	↑*DMN ↔ right CN*	↑*AN ↔ right CN*	↑**right CN** ↑*right CN ↔ DMN/AN*	–	–	↑**right CN** ↑*right CN ↔ DMN/AN*	–
	Nakamura et al. (150)	–	–	–	–	–	–	–
	Nemati et al. (151)	–	–	–	–	–	–	–
	Nugent et al. (152)	–	*↓right AMYG ↔left insulo-temporalareas* ↓ sgACC ↔ precentral gyrus	–	–	–	–	*↓ right AMYG *↔ left* insulo-temporal areas*
	Reed et al. (153)	↓ l**eft LTL**	–	–	↓**right DLPFC**	↓**dACC**	↑**CBM**	–
	Reed et al. (154)	↓**mPFC** ↓**PCC/PCu**	↓**INS**	–	–	↓**AI**	–	↓**INS**
	Roy et al. (155)	–	–	↑ right NAc	–	–	–	–
	Sahib et al. (156)	↑***PCC*** ↑ ***PCu***	*↓**right INS*** *↓**bilateral HPC***	–	–	*↓**right AI***	–	*↓**right INS***
	Sahib et al. (157)	↓**IPL** ↓**within-network (right)**	–	–	↓**DLPFC** ↓**SPL** ↓**within-network (right)**	↓**within-network (right)**	↑**SMA** ↓**right CBM**	–
	Sahib et al. (158)	–	–	*↓ BG ↔ CBM*	–	*↓ SN ↔ CBM*	*↓ CBM ↔ SN* *↓ BG ↔ CBM*	–
	Salvadore et al. (159)	–	–	–	–	–	–	–
	Salvadore et al. (160)	–	–	–	–	–	–	–
	Siegel et al. (161)	*↓ within-network* *↓ DMN ↔ bilateral sgACC*	*↓ bilateral sgACC ↔ DMN* ↑*sgACC ↔ bilateral caudal ACC* ↑*sgACC ↔ bilateral AI* *↓ within limbic system (AMYG, anterior TH, and anterior HPC, NAc)*	*↓ within limbic system (AMYG, anterior TH, and anterior HPC, NAc)*	–	↑*bilateral AI ↔ sgACC*	*↓ within limbic system (AMYG, anterior TH, and anterior HPC, NAc)*	*↓ within limbic system (AMYG, anterior TH, and anterior HPC, NAc)*
	Sterpenich et al. (162)	–	↓**AMYG** ↓**INS (emotion task)** ↑**INS (reward task)** ↑ **mOFC**	↑**mOFC** ↑**ventral striatum** ↑ **VTA/SNc**	–	↓**dACC** ↑**AI (reward task)**	↑**ventral striatum** ↑ **VTA/SNc**	↓**AMYG** ↓**INS (emotion task)** ↑**INS (reward task)**
	Thai et al. (163)	↓**PCC/PCu**	↓**left and right HPC/left and right AMYG/ACC** ↑**right HPC (congruent positive)**	↓**left and right NAc**	**–**	**–**	**–**	↓**left and right HPC/left and right AMYG/ACC**
	Tiger et al. (164)	–	↑ HPC*	–	–	–	–	–
	Vasavada et al. (165)	–	*↓ AMYG ↔ left CEN (24 h)* *↑ right HPC ↔ left CEN (24 h)* *↑ AMYG ↔ left CEN (24–48 h)* *↑ right AMYG ↔ right CEN* *↑ right HPC ↔ left CEN (24–48 h)* *↓ left AMYG ↔ SN (24–48 h)*	–	*↓ left CEN ↔ AMYG (24 h)* *↑ left CEN ↔ right HPC (24 h)* *↑ left CEN ↔ AMYG (24–48 h)* *↑ right CEN ↔ right AMYG* *↑ left CEN ↔ right HPC (24–48 h)*	*↓ SN ↔ left AMYG (24-48 h)*	–	*↓ AMYG ↔ left CEN (24 h)* *↑ AMYG ↔ left CEN (24–48 h)* *↑ right AMYG ↔ right CEN* *↓ left AMYG ↔ SN (24–48 h)*
	Wang et al. (166)	–	–	–	–	–	–	–
Memantine	Kilpatrick et al. (167)	–	–	–	–	–	–	–
D-cycloserine	Cole et al. (168)	–	–	–	–	–	–	–
Nitrous oxide	–	–	–	–	–	–	–	–
4-chlorokynurenine	Park et al. (169)	–	–	–	–	–	–	–
Lanicemine	Abdallah et al. (133)	–	–	–	–	–	–	–
	Downey et al. (137)	↑**rACC** ↑**right PCC**	↑**vACC/sgACC**	↑**bilateral CN**	–	↑**bilateral dACC**	↑**bilateral TH** ↑**bilateral CN** ↑**bilateral BS**	↑**bilateral BS**

*ACC, anterior cingulate cortex; AI, anterior insula; AMYG, amygdala; AN, ventromedial affective network; BG, basal ganglia; BS, brainstem; CAN, frontovagal central autonomic network; CBM, cerebellum; CEN, frontoparietal central executive network; CN, caudate nucleus; dACC, dorsal anterior cingulate cortex; DMN, default mode network; DLPFC, dorsolateral prefrontal cortex; DMPFC, dorsomedial prefrontal cortex; HPC, hippocampus; INS, insula; IPL, inferior parietal lobule; LHb, lateral habenula; LTL, lateral temporal lobe; M1, primary motor cortex; mOFC, medial orbitofrontal cortex; mPFC, medial prefrontal cortex; MTL, medial temporal lobe; NAc, nucleus accumbens; PCC, posterior cingulate cortex; PCu, precuneus; PFC, prefrontal cortex; pgACC, pregenual anterior cingulate cortex; Pu, putamen; rACC, rostral anterior cingulate cortex; RN, ventral frontostriatal reward network; S1, primary somatosensory cortex; sgACC, subgenual anterior cingulate cortex; SMA, supplementary motor area; SMN, frontocerebellar sensorimotor network; SN, anterior cinguloinsular salience network; SNc, substantia nigra pars compacta; SPL, superior parietal lobule; TH, thalamus; vACC, ventral anterior cingulate cortex; VTA, ventral tegmental area*.

*Asterisks, serotonin (5-HT_1B_) receptor binding; bold, blood-oxygen-level-dependent (BOLD) response; bold italics, cerebral blood flow; dotted underline, glucose metabolism; double underline, entropy; italics, functional connectivity; italics underlined, magnetoencephalographic (MEG) connectivity; no formatting, global brain connectivity (GBC). ↑, increased; ↓, decreased; ↔, functional connectivity between two brain regions*.

**Table 4B:** Intrinsic connectivity network biomarkers predicting antidepressant response to glutamatergic interventions.

**Glutamatergic compound**	**Reference**	**DMN**	**AN**	**RN**	**CEN**	**SN**	**SMN**	**CAN**
Ketamine	Abdallah et al. (131)	↑ left MTL	↑ left INS	↑ bilateral CN	↑ bilateral DLPFC	↑ left AI	↑ bilateral CN	↑ left INS
	Abdallah et al. (132)	–	↑ VMPFC	↑ VMPFC	–	–	–	–
	Abdallah et al. (133)	–	↓ VMPFC	↓ VMPFC	↑ DLPFC	–	–	–
	Carlson et al. (134)	↑right _ _ _LTL _ _↓right _ _ _ _MTL_ ↓ left _ _IPL	↓right _ _ _ _HPC _ ↓ vACC	–	–	–	↑left _ _ _ _CBM	–
	Chen et al. (135)	–	*↓ right vACC/sgACC ↔ left dACC (suicidal ideation)*	–	*↑ right DLPFC ↔ left SPL (suicidal ideation)*	*↓ left dACC ↔ right vACC/sgACC (suicidal ideation)*	–	–
	Chen et al. (136)	–	–	*↓ executive BG ↔ superior frontal gyrus*	*↓ superior frontal gyrus ↔ executive BG*	–	*↓ executive BG ↔ superior frontal gyrus*	–
	Downey et al. (137)	↑**rACC**	↑**sgACC**	–	–	↑**dACC**	–	–
	Evans et al. (138)	–	–	–	–	–	–	–
	Gärtner et al. (139)	–	*↑ sgACC ↔ right DLPFC*	–	*↑ right DLPFC ↔ sgACC*	–	–	–
	Gilbert et al. (140)	–	*↓INS ↔ ACC*	–	–	*↓AI ↔ dACC*	–	*↓INS ↔ ACC*
	Kraus et al. (141)	–	–	–	–	–	–	–
	Lally et al. (142)	–	↑right _ _ _ _HPC _ _ _ _ _ _ _ _(anhedonia) _ ↓ right _ _ _ _mOFC _ _ _ _ _ _ _ _(anhedonia)	↓right _ _ _ _ _mOFC _ _ _ _ _ _ _ _(anhedonia)	–	↑dACC _ _ _ _ _ _ _ _(anhedonia)	–	–
	Li et al. (143)	–	–	–	–	–	–	–
	Loureiro et al. (144)	–	↓**right AMYG (anhedonia)** ↑**right INS**	–	↑**right DLPFC (fearful cue)** ↓**right DLPFC (happy cue; anhedonia)**	↑**right AI**	–	↓**right AMYG (anhedonia)** ↑**right INS**
	Loureiro et al. (145)	–	–	–	*↓ CEN ↔ CBM*	–	*↓ CBM ↔ CEN* *↓ CBM ↔ SMN*	–
	McMillan et al. (146)	–	–	↑**right INS**	–	–	↓**S1**	↑**right INS**
	Mkrtchian et al. (147)	–	–	↑*dorsal CN ↔ right ventrolateral PFC (anhedonia)* *↑ dorsal CN ↔ pgACC (anhedonia)*	–	–	↑*dorsal CN ↔ right ventrolateral PFC (anhedonia)* *↑ dorsal CN ↔ pgACC (anhedonia)*	–
	Morris et al. (148)	–	↑**sgACC (anhedonia)**	–	–	–	–	–
	Murrough et al. (149)	↑*DMN ↔ right CN*	↑*AN ↔ right CN*	↑*right CN ↔ ACC*	–	–	↑*right CN ↔ ACC*	–
	Nakamura et al. (150)	–	*↓ right sgACC ↔ right AMYG*	–	–	–	–	*↓ right AMYG ↔ right sgACC*
	Nemati et al. (151)	↓*DMN ↔ SMN*	–	*↓ GPu SC within* ↑ GPu SC ↔ rest of brain	↓*within-network* ↑ with rest of brain	↓ SN ↔ SMN	↓*within-network* *↓ GPu SC within* ↑ GPu SC ↔ rest of brain ↓*SMN ↔ SN* ↓*SMN ↔ DMN*	–
	Nugent et al. (152)	–	–	–	–	–	–	–
	Reed et al. (153)	↓**PCu (angry cue)** ↑**PCu (happy cue)**	↓**AMYG (angry cue)** ↓**left parahippocampal gyrus (angry cue)** ↑**AMYG (happy cue)** ↑**left parahippocampal gyrus (happy cue)**	–	↑**DLPFC (happy cue)** ↓**DLPFC (angry cue)**	↑**dACC (happy cue)** ↓**dACC (angry cue)**	–	↓**AMYG (angry cue)** ↑**AMYG (happy cue)**
	Reed et al. (154)	–	–	–	–	–	–	–
	Roy et al. (155)	–	–	↑ right NAc	–	–	–	–
	Sahib et al. (156)	–	–	–	–	–	–	–
	Sahib et al. (157)	–	–	–	↓**within-network**	–	↓**SMA**	–
	Sahib et al. (158)	–	–	↑*BG ↔ CBM*	–	↑*SN ↔ CBM*	↑*BG ↔ CBM* ↑*CBM ↔ SN*	–
	Salvadore et al. (159)	↑rACC	↓right AMYG	–	–	–	–	↓right AMYG
	Salvadore et al. (160)	–	*↓left AMYG ↔ pgACC*	–	–	–	–	*↓left AMYG ↔ pgACC*
	Siegel et al. (161)	*↓ within-network*	–	–	–	–	–	–
	Sterpenich et al. (162)	–	↑**mOFC**	↑**mOFC** ↑**ventral striatum** ↑ **VTA/SNc**	–	–	↑**ventral striatum** ↑ **VTA/SNc**	–
	Thai et al. (163)	↓**PCC/PCu**	↓**left and right HPC/left and right AMYG/ACC** ↑**right HPC (congruent positive)**	↓**left and right NAc**	–	–	–	↓**left and right HPC/left and right AMYG/ACC**
	Tiger et al. (164)	–	–	↓ ventral striatum*	–	–	↓ ventral striatum*	–
	Vasavada et al. (165)	–	*↑ right HPC ↔ left CEN (anhedonia)*	–	*↑ left CEN ↔ right HPC (anhedonia)*	–	–	–
	Wang et al. (166)	*↓ left PCu ↔ left LHb* *↓ left PCu ↔ right LHb* *↓ bilateral angular gyrus ↔ right LHb*	–	*↓ left LHb ↔ left PCu* *↓ right LHb ↔ left PCu* *↓ right LHb ↔ bilateral angular gyrus*	–	–	–	–
Memantine	Kilpatrick et al. (167)	*↑ within-network*	–	–	–	–	–	–
D-cycloserine	Cole et al. (168)	–	–	–	–	–	–	–
Nitrous oxide	–	–	–	–	–	–	–	–
4-chlorokynurenine	Park et al. (169)	–	–	–	–	–	–	–
Lanicemine	Abdallah et al. (133)	–	–	–	↑ DLPFC	–	–	–
	Downey et al. (137)	↑**rACC**	↑**sgACC**	–	–	↑**dACC**	–	–

*ACC, anterior cingulate cortex; AI, anterior insula; AN, ventromedial affective network; BG, basal ganglia; CAN, frontovagal central autonomic network; CBM, cerebellum; CEN, frontoparietal central executive network; CN, caudate nucleus; dACC, dorsal anterior cingulate cortex; DLPFC, dorsolateral prefrontal cortex; DMN, default mode network; GPu SC, globus pallidus-putamen subcortical; HPC, hippocampus; INS, insula; IPL, inferior parietal lobule; LHb, lateral habenula; LTL, lateral temporal lobe; MTL, medial temporal lobe; NAc, nucleus accumbens; PCu, precuneus; pgACC, pregenual anterior cingulate cortex; rACC, rostral anterior cingulate cortex; RN, ventral frontostriatal reward network; S1, primary somatosensory cortex; sgACC, subgenual anterior cingulate cortex; SMA, supplementary motor area; SMN, frontocerebellar sensorimotor network; SN, anterior cinguloinsular salience network; SNc, substantia nigra pars compacta; SPL, superior parietal lobule; vACC, ventral anterior cingulate cortex; VMPFC, ventromedial prefrontal cortex; VTA, ventral tegmental area*.

*Asterisks, serotonin (5-HT_1B_) receptor binding; bold, blood-oxygen-level-dependent (BOLD) response; bold underlined, magnetoencephalographic (MEG) activity; dotted underline, glucose metabolism; double underline, entropy; italics, functional connectivity; italics underlined, magnetoencephalographic (MEG) connectivity; no formatting, global brain connectivity (GBC). ↑, increased; ↓, decreased; ↔, functional connectivity between two brain regions*.

## Intrinsic Connectivity Network Biomarkers of Ketamine Antidepressant Response

The discovery of rapid antidepressant effects of ketamine, an arylcyclohexylamine derivative and a non-selective NMDA-R antagonist, has revolutionized the field of investigational treatments for depression. The first data demonstrating the antidepressant properties of racemic ketamine were published by Berman et al. (14). Since then, several meta-analyses have confirmed its efficacy for depression, including TRD (170–172). Ketamine is believed to antagonize the NR2B subunit of an NMDA-R situated on GABAergic interneurons. This results in local circuit disinhibition and a consequent increase in the release of glutamate and brain-derived neurotrophic factor (BDNF), triggering the upregulation of AMPA-R expression and the stimulation of non-NMDA-R-mediated glutamatergic neurotransmission (19, 173). Molecularly, these processes modulate the second messenger intracellular signaling pathways involving mammalian target of rapamycin (mTOR), Calcium/Calmodulin-Dependent Protein Kinase II (CaMKII), the Eukaryotic Elongation Factor 2 Kinase (eEF2K) pathway, methyl-CpG-binding protein 2 (MeCP2) phosphorylation, and brain glycogen synthase kinase-3 (GSK3) (174–179). At a systems level, these molecular cascades stimulate synaptic plasticity and synaptogenesis, leading to the reorganization of neural networks in adaptation to the inputs from the environment (19, 180). Behaviorally, while the rapid antidepressant effects appear to be evident from clinical observations, increased synaptogenesis could contribute to long-term changes in ICNs and, as a corollary, a sustained antidepressant response.

### Global Brain Connectivity and Connectome Fingerprints

While to date fMRI research still largely remains hypothesis-driven, primarily relying on conventional seed-based methods and an *a priori* selection of regions of interest, more recent advances in the field have highlighted the importance of data-driven assessments of whole-brain functional connectome without the need for seed selection and independent component analysis (22, 181). Applications of graph theory approaches have made it possible to identify hubs, or nodes (brain regions) that have a significantly larger number of edges in comparison to other nodes within a network, making such regions globally connected within the brain (182, 183). Anatomical (184) and functional (182, 185) whole-brain connectivity methods have generally agreed that nodes within the DMN and CEN, the two large-scale ICNs anti-correlated during functional task performance and uncorrelated at rest (186, 187), possess among the highest global brain connectivity (GBC), which reflects the hierarchical organization of intrinsic functional architecture of the brain and highlights the role of these hubs in coordinating a wide array of cognitive and behavioral outcomes (188).

GBC, or functional connectivity strength, is a correlation-based approach that constructs a three-dimensional map of an fMRI scan by calculating the correlation of the time series of each gray matter (GM) voxel with all other GM voxels in the brain, which are subsequently transformed into Fisher z-scores and averaged (141, 182). Both unweighted (185) and weighted (189) GBC methods have been developed, both of which are able to reveal a non-directional functional connectivity profile of high-degree network nodes and enable the identification of globally connected or disconnected brain regions in a data-driven fashion, without the influences of between-subjects or intraregional spatial variations in connectivity patterns (190). Global signal regression is frequently used in GBC analyses to preprocess the fMRI global signal and remove residual motion artifacts and physiological noise (187). GBC with global signal regression (GBCr) has been actively explored as a potentially robust and reproducible ICN biomarker, where GBCr values have been used to identify major brain ICNs (182) and explore ICN alternations in psychiatric disorders characterized by chronic stress and underlying glutamate synaptic homeostasis pathology, including bipolar disorders, obsessive-compulsive disorder, post-traumatic stress disorder, psychosis, and MDD (131, 132, 141, 190–200). In depression, specifically, several GBC studies have revealed reduced global connectivity in the core nodes of the CEN and DMN, including the regions of the medial and lateral PFC, as well as the PCC/Precuneus (PCu) (131, 132, 141, 192, 193, 198–200). Theoretically, these findings have been discussed in the context of stress-induced chronic glutamate activation, excitotoxicity, and NMDA-R hypofunction—the processes hypothesized to subsequently lead to reduced synaptic strength, synaptic dysconnectivity, and, as a corollary, reduced GBC in high-degree nodes (131, 132). In light of this evidence, GBCr as a marker is thought to be positively correlated with the levels of glutamate in the synapse, and the findings confirming the reduced GBCr in MDD support the hypoglutamatergic hypothesis behind the etiology of the disorder.

**Figure 4:**
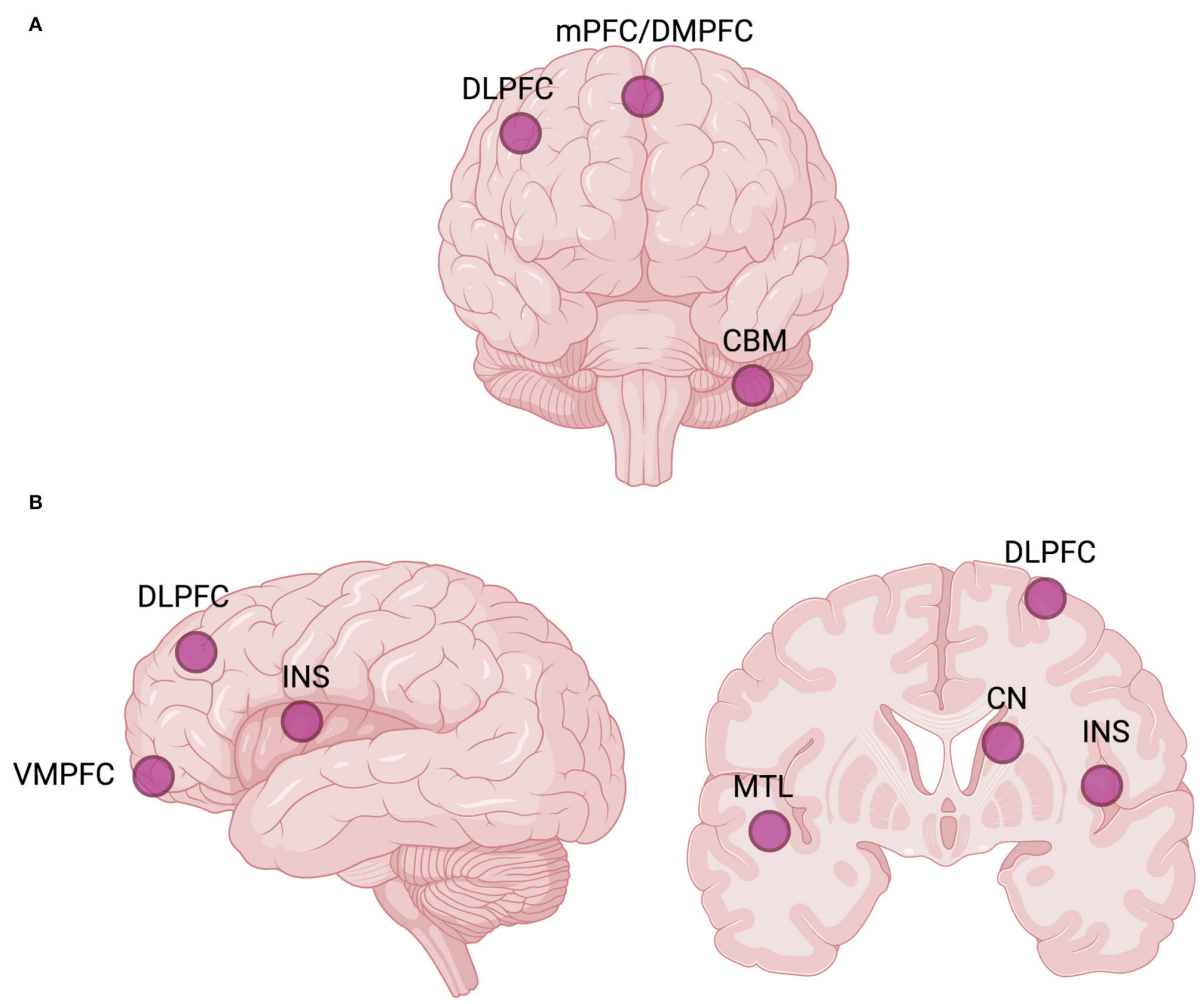
Global brain connectivity (GBC) hubs, or network nodes interconnected with the rest of the brain, may be investigated as potential biomarkers of rapid antidepressant response to ketamine and other glutamatergic interventions (131–133). **(A)** Commonly reported GBC hubs targeted by ketamine. **(B)** Commonly reported GBC hubs implicated in predicting rapid antidepressant treatment response to ketamine. The laterality of hubs is not considered. Created with BioRender.com, RRID:SCR_018361. CBM, cerebellum; CN, caudate nucleus; DLPFC, dorsolateral prefrontal cortex; DMPFC, dorsomedial prefrontal cortex; INS, insula; mPFC, medial prefrontal cortex; MTL, medial temporal lobe; VMPFC, ventromedial prefrontal cortex.

Ketamine has been repeatedly shown to increase GBCr in healthy individuals (194, 201, 202). Among MDD participants, ketamine appears to increase DLPFC (i.e., the CEN node) and mPFC/dorsomedial PFC (DMPFC, the dorsal nexus) GBCr during infusion and at 24 h post-treatment (131–133), but not at 48 h post-treatment (141) (Figure 4). Ketamine was also reported to significantly reduce the GBCr in the cerebellum (131). However, ketamine treatment failed to alter ventromedial PFC (VMPFC) GBCr 24 h post-treatment in TRD patients, which is a cluster associated with the AN (132). GBCr marker also appears to be correlated with ketamine treatment response. For instance, ketamine responders show elevated GBCr in the lateral PFC, caudate, and insula compared to non-responders (131). VMPFC GBCr was also predictive of treatment response, even though ketamine did not have any significant effect on the VMPFC connectivity (132, 133). Even though ketamine failed to normalize VMPFC GBCr, it nevertheless was predictive of treatment response. On the other hand, lanicemine (AZD6765), a low-trapping NMDA-R antagonist, was demonstrated to reduce mPFC GBCr associated with the DMN (132), while other reports have identified no significant effects of lanicemine on GBCr (133). To interpret these findings, it was hypothesized that MDD, as a brain network pathology, was characterized by increased within-network connectivity of PFC-subcortex ICNs and decreased between-network connectivity of the PFC with the rest of the brain (131). Post-ketamine NMDA-R blockade and the resultant surge in glutamate were suggested to lead to the normalization of connectivity dysfunction through an induced elevation in between-network connectivity, which, at the behavioral level, might underly the change from rumination and withdrawal to exploratory and externally oriented behaviors. To date, nodes within the PFC remain the primary points of interest for glutamatergic GBC studies, where the effects of ketamine and other glutamatergic treatments on the GBC of major hubs still need more robust characterization, further validation, and replication.

The two major advantages of the GBC approach are the ability to obtain the measurements of nodal strength, a fundamental network topology measure, as well as to conduct whole-brain connectivity analysis without an *a priori* seed selection (151). However, GBC is unable to reveal which specific edges within a network drive the pathology, and it does not capture network connectivity changes in the opposing directions, such as shifts from within-network connectivity to between-network connectivity. An emerging method that potentially addresses these limitations is profiling the functional connectivity of all brain networks, another data-driven approach termed functional connectome fingerprinting. This approach combines network-restricted strength (NRS) methods with classical connectome-based predictive modeling (CPM) (203, 204). The NRS predictive model is used not to evaluate the strength of predictions but rather to identify the functional connectivity signature, or a unique connectome fingerprint (CFP), that is significantly associated with an outcome of interest (205). Connectome fingerprinting has been successfully tested in predicting behavior and personality traits in healthy individuals (206, 207) and patients with various neuropsychiatric conditions (31, 151, 208–212).

For depression, studies have identified unique CFPs that predicted suicidal ideation (211), treatment response to the serotonin selective reuptake inhibitor (SSRI) sertraline (151, 212), and general antidepressant response independent of treatment modality (31). A study by Nemati et al. (151) has identified a unique CFP predictive of antidepressant response to sertraline, which had particular relevance to glutamatergic treatment modalities, notably ketamine. Identified 1 week after initiation of the SSRI treatment, this CFP was comprised three connectivity patterns, including (1) reduced within-network connectivity in the CEN, SMN, visual network, and globus-pallidus-putamen subcortical (GPu SC) network; (2) elevated connectivity between the nodes of the CEN and the rest of the brain, as well as between the nodes of the GPu SC network and the rest of the brain; and (3) diminished connectivity of the nodes within the DMN and SN with the sensorimotor and visual networks but elevated connectivity with higher association areas, indicative of an early shift toward enhanced executive control. All three CFP patterns predicted response to ketamine at 24 h compared to lanicemine (active control) but failed to predict response to ketamine relative to the placebo.

A more recent study (212) has identified a CFP associated with intravenous infusion of ketamine, where NRS predictive models were applied to the data from healthy participants and subsequently validated in a cohort of 200 MDD patients. At week 1 post-infusion, ketamine was associated with reduced within-network connectivity in the CEN, SMN, and visual network, as well as with elevated connectivity between the nodes of the CEN and the rest of the brain. Among MDD patients, these observed ketamine-induced connectivity changes predicted antidepressant response to sertraline at week 8. The findings were interpreted as a shift from internal (i.e., within-network) to external (i.e., between-network) connectivity within the CEN caused by a glutamate surge, indicative of a therapeutic effect of ketamine on top-down executive control that is impaired in depression (213). As such, both increases and decreases in synaptic connectivity were required to achieve a neurobiologically meaningful rapid antidepressant response at the ICN level (212). Interestingly, it was hypothesized that other glutamatergic treatment interventions that affect connectivity locally, as opposed to globally, may not lead to sustained normalization of ICNs and robust rapid antidepressant effects. This might partially explain why rapastinel, an NMDA-R allosteric modulator with glycine-site partial agonist properties, failed in TRD clinical trials despite its ability to increase PFC synaptic connectivity (214, 215).

### Default Mode Network

The DMN is comprised of the mPFC/rACC, PCC, PCu, and bilateral inferior parietal lobule (IPL), representing a set of regions activated during task-negative control conditions, or “at rest” (62). The DMN is an interconnected and anatomically defined ICN that can be further separated into at least three major interacting subnetworks with specific functions: a midline “core” subnetwork (i.e., mPFC and PCC) that is involved in self-referential processing but that gets consistently activated for all DMN-relevant functions, the dorsal medial subnetwork [i.e., the PFC, PCC, angular gyrus, temporoparietal junction (TPJ), and temporal pole] involved in mentalizing and conceptual processing, and the medial temporal subnetwork (i.e., IPL, temporal cortex) involved in constructive mental simulation and episodic/contextual retrieval (216, 217). Most commonly, the activity within the DMN has been reported for processes that permit an individual to construct personal meaning from salient information, including autobiographical memory retrieval, theory of mind, spatial navigation and cognition, personal decision-making, mind-wandering, remembering the past, and imagining the future (218). Depression is associated with increased nodal centralities (65) and elevated within-network DMN connectivity at rest (219) and during engagement in externally focused thought (220), both of which manifest in excessive self-referential processing and maladaptive rumination. Moreover, stronger pre-treatment connectivity between posterior (PCC) and anterior nodes (mPFC) of the DMN correlates with antidepressant response to pharmacotherapy and electroconvulsive therapy (23), and a recent coordinate-based meta-analysis has concluded that baseline within-network DMN connectivity at rest predicts antidepressant response regardless of treatment modality (221).

A growing body of studies indicates that DMN connectivity can be a potential target of glutamatergic interventions for depression and supports its role in predicting rapid antidepressant response. Evidence pooled from ^1^H-MRS studies agrees that ketamine transiently increases GLX levels in the mPFC of MDD individuals (222), which is a central hub within the midline core subnetwork. However, despite possible increases in glutamate levels, neuroimaging studies generally report that glutamatergic compounds decrease DMN activity and connectivity, congruent with antidepressant action of other treatment modalities on the DMN pathological hyperfunctioning. For instance, Evans et al. have shown normalization of inappropriately increased insula-PCC functional connectivity among MDD patients 48 h after ketamine infusion, which was, however, reversed after 10 days (138). In another study conducted among TRD patients who received a continuous 96 h infusion of ketamine, responders showed a greater decrease in DMN connectivity than non-responders (161).

Additionally, evidence suggests that glutamatergic treatments potentially normalize DMN hyperactivity in depression not only at rest but also during the core manifestations of MDD psychopathology, such as affective dysregulation. Using an emotional processing task, Reed et al. (154) showed that ketamine decreased the activity of the core DMN hubs, including the mPFC and PCC/PCu. This was further supported by the evidence of decreased DMN activity in response to incongruent emotional words and faces among TRD adults who received six ketamine infusions prior to the scan session (163). Interestingly, this was predictive of treatment response (163). Moreover, the predictive power of DMN changes was also shown in a resting-state simultaneous EEG/fMRI study (146) conducted among MDD patients who received ketamine. In that study, BOLD signal variance in the PCC was explained by high gamma power, and responders exhibited a smaller change in BOLD signal relative to non-responders. Given that the intranetwork connectivity within the DMN is most likely facilitated by cortico-cortical glutamatergic connections, DMN hyperconnectivity represents a viable target for glutamatergic treatment options that could possibly be directly modulated through therapeutically induced changes in glutamate levels.

### Ventromedial Affective Network

The medial orbitofrontal cortex (mOFC)/VMPFC, rostroventral portion of the ACC, including the sgACC, and limbic regions, including AMYG, HPC, and insula, form a ventral network implicated in emotional processing and regulation, known as the AN (68–70). Individual nodes of the VMPFC are involved in the generation (sgACC) and regulation (mOFC) of negative affect (223, 224), and the sgACC, a core node of the AN, was one of the earliest neural markers of MDD and antidepressant treatment response (219, 224, 225). Hyperactivity of the AMYG and sgACC, as well as increased within-network AN connectivity, have been consistently reported in depression (226). From a functional standpoint, increased sgACC activity is linked with the generation of negative mood states (224), while increased VMPFC activity is associated with a successful top-down inhibition thereof (227). Neuroimaging findings have highlighted changes in the sgACC volume, function, and connectivity that can predict treatment response to a variety of antidepressant interventions and may be utilized to guide treatment selection (23). Further, improved response to antidepressants has been associated with a stronger VMPFC-AMYG (228) and AMYG-ACC (229) connectivity, which supports the notion that cortical nodes exert diminished glutamatergic top-down regulation over the AMYG activity in MDD (226).

The role of sgACC in predicting antidepressant response to glutamatergic interventions has been greatly supported by the resting-state and task-based neuroimaging literature of the past decade. Lower sgACC-AMYG (150) and sgACC-right lateral PFC (139, 152) functional connectivity has been shown to predict treatment response to ketamine. Additionally, ketamine was demonstrated to increase the sgACC-right lateral PFC connectivity (139). In a similar fashion, a study by Downey et al. showed that both ketamine and lanicemine increased the BOLD signal in the sgACC, which predicted improvements in MDD symptoms at 24 h and 1-week post-treatment (137). While ketamine evoked greater activations in sgACC BOLD signals than lanicemine, there was no significant antidepressant response to either intervention compared to placebo. Nevertheless, the brain network model of depression views sgACC as an aberrantly hyperactive node at baseline, and several studies (148, 230) that explored the effect of ketamine on sgACC supported this postulate, demonstrating a reduction in sgACC hyperactivity that was in line with the effect of other antidepressant treatment modalities (224, 225). Therefore, given the conflicting findings concerning the effects of ketamine on sgACC BOLD response, it is uncertain whether sgACC functionality in MDD could be directly coordinated *via* glutamatergic inputs.

Nodes of the AN and within-network AN connectivity have also been shown to be modulated by ketamine. Fluorodeoxyglucose (^18^F-FDG) PET imaging *in vivo* metabolism studies showed evidence of altered metabolism in the AMYG (134), insula (134), and HPC (231) post-ketamine, and regional decreases in the cerebral blood flow have been observed in the right insula and bilateral HPC in TRD patients (156). Recently, Siegel et al. have demonstrated that MDD participants display a treatment-dependent decrease in AN hyperconnectivity after ketamine administration (161), with reported results involving altered functioning of the AMYG, anterior TH, and anterior HPC. A MEG study also reported that ketamine administration induced a decrease in connectivity between the AMYG and insulo-temporal areas (152), which further supports the within-network AN connectivity reduction following an NMDA-R blockade. Activity within the nodes of the AN also appears to predict antidepressant response. Evidence from early MEG studies suggests that increased baseline pregenual ACC (pgACC)/rACC activity and decreased AMYG activity in response to fearful stimuli predict antidepressant response to ketamine 4 h post-administration (159). Moreover, TRD patients with the deactivation of pgACC during a working memory task and with the lowest coherence between pgACC and left AMYG showed the greatest improvement in depression post-ketamine (160). Further, using an attentional bias probe task, Reed et al. reported that improvement in depression post-ketamine was associated with diminished activity within the AMYG, parahippocampal gyrus, and cingulate gyrus during angry cues but with elevated activity of these regions during happy cues (153). Moreover, ketamine reduces AMYG hyperactivity, which also correlates with treatment response. In the most recent study that explored the AN involvement in ketamine response, treatment-induced post-treatment decreases in AMYG BOLD activity during the presentation of both negative and positive emotional face stimuli were correlated with improvements in depression and anhedonia (144). Baseline AMYG activity, however, did not have significant predictive power, and, in fact, a case report by Scheele et al. (232) documented the response to ketamine in a TRD patient with bilateral AMYG damage, suggestive of the fact that AMYG activity might not be necessary for the antidepressant response to glutamatergic treatments (150).

### Ventral Frontostriatal Reward Network

The RN, comprised of VMPFC/mOFC, striatum (NAc, caudate, putamen), and VTA/SNc, is a ventral network that contributes to reward processing, reward prediction, and reward-based reversal learning (233, 234). While primary reward anticipation and evaluation are managed *via* dopaminergic bottom-up projections from the striatum, glutamatergic top-down inputs from VMPFC/mOFC over the striatum are functionally related to reward learning and adaptive decision-making (235–238). With the core node in the NAc, the within-network connectivity of the RN appears to be diminished in MDD, which accounts for anhedonia and avolition (71–74). Increased frontostriatal connectivity has been shown to be associated with better clinical outcomes after a course of an antidepressant, possibly suggesting that RN connectivity might predict treatment response (239). Nevertheless, findings concerning the predictive value of reward-based neural markers of antidepressant response are mixed (240–244), and, since clinical neuroimaging studies in this domain are still in relatively early stages, translational gaps in the anhedonia literature remain (245).

Ketamine is characterized by well-known anti-anhedonic effects: increases in glucose metabolism within implicated brain areas of the RN, including the ventral striatum, have been previously reported (142, 231, 246). One study on TRD patients who received a single dose of ketamine revealed that increased activity of the reward-related brain regions, including the mOFC, ventral striatum, and VTA/SNc, is associated with improvement in depression symptoms (162). Ketamine leads to at least partial recovery of the diminished reward function, and while the precise molecular mechanisms behind these effects are unknown, existing evidence indicates that ketamine can increase the release of striatal dopamine (247, 248) and affect dopaminergic function (249), most likely through glutamatergic inputs from the VMPFC/mOFC. Similarly, ketamine increases GBCr in the striatum (131) and augments striatal response during emotional processing (149). Recently, in a study among TRD adolescents who received six ketamine infusions, Roy et al. (155) showed that the reduction in depressive symptoms was associated with increased NAc entropy, a measure of neural flexibility. Further, ketamine responders showed a greater increase in NAc entropy than non-responders, where neural flexibility of RN nodes was predictive of rapid antidepressant response. Increased serotonin receptor binding in the ventral striatum was also shown to predict treatment response (164). Other RN connectivity markers of antidepressant response to ketamine include the decreased connectivity of the bilateral superior frontal cortex with the executive region of the striatum (136) and the increased connectivity of the caudate with the ACC (147, 149). The most recently identified marker of ketamine treatment response is a reported increase in the RN within-network connectivity in TRD patients, where the improvement in anhedonia scores was associated with increased connectivity between the dorsal caudate and ventrolateral PFC, as well as between the dorsal caudate and pgACC (147).

### Frontoparietal Central Executive Network

The frontoparietal regions of the CEN display a significant overlap with networks involved in attention and top-down control, including the dorsal attention network (DAN) and cognitive control network (CCN), and therefore, in this review, we define the CEN as comprised of the DLPFC, frontal eye fields, superior parietal lobule (SPL), and intraparietal sulcus (250, 251). The CEN involvement is implicated in a wide array of behaviors that represent higher-order cognitive and executive functioning, including action planning, working memory, sustained attention, decision-making and problem-solving in the context of goal-directed behavior, behavioral inhibition, and cognitive flexibility (251). One of the hallmark characteristics of depression is a failure of effective cognitive control over emotional processing, which is most likely attributed to diminished within-network CEN connectivity (252). DLPFC activity during cognitive and working memory tasks, in particular, represents a promising candidate biomarker of antidepressant response to pharmacotherapy and repetitive transcranial magnetic stimulation (rTMS), with a well-established base of evidence (23).

Ketamine has been demonstrated to increase the functional connectivity of the CEN (132, 133, 139, 147, 212), possibly due to direct increases in PFC glutamate levels (213). DLPFC activity and connectivity, in particular, appear to be viable candidate biomarkers of rapid antidepressant response. However, it should be noted that CEN connectivity under glutamatergic interventions differs between resting-state and functional tasks (201), and, therefore, activity and connectivity results may greatly depend upon study settings. Nevertheless, several studies have shown the association between CEN and treatment response to glutamatergic treatments in depression. Sahib et al. (157) showed that ketamine induced a significant decrease in the BOLD response of CEN nodes responsible for response inhibition, including the DLPFC, among TRD patients who performed a Go/NoGo task. Moreover, ketamine remitters enrolled in that study had lower baseline BOLD activity in the CEN nodes than non-remitters, which indicates the CEN's potential to predict treatment response to ketamine. Further, during the resting state, Chen et al. (135) showed a significant decrease in functional connectivity between the right DLPFC and right frontal cortex 48 h after a single ketamine infusion in TRD participants. This paralleled an increase in the within-network CEN connectivity, notably between the right DLPFC and left superior parietal cortex, that was positively correlated with the reduction in suicidal ideation. Lastly, during an affective processing task, greater BOLD signals in the DLPFC in response to fearful stimuli post-ketamine were correlated with antidepressant response, while reduced DLPFC BOLD signals in response to happy stimuli—with improvements in anhedonia (144).

### Anterior Cinguloinsular Salience Network

The SN was first identified by Seeley et al. (253) as a network comprised of dACC and AI, the activity of which was correlated with anxiety ratings. The nodes and functions of SN tend to overlap with the ventral attention network (VAN), implicating TPJ, although the latter also involves frontoparietal brain regions. Subsequently, the four basic mechanisms associated with the SN have been defined: (1) identification of relevant external information or detection of salient stimuli, (2) switching of the focus of attention to salient stimuli, (3) facilitation of autonomic response to salient stimuli *via* projections to the nodes within the CAN, and (4) initiation of goal-directed behaviors *via* projections to the premotor and motor cortex from the dACC (254). Hypoactivity of the SN in depression has been associated with anhedonia and poor incentive salience (64, 75–77), and pre-treatment activity of dACC and AI during emotional processing have been identified as predictors of antidepressant response (20, 23).

^18^F-FDG PET studies have highlighted the role of the dACC in mediating ketamine antidepressant effects. Ketamine appears to increase the activity and glucose metabolism of the dACC, which has been associated with improvements in anhedonia, the core MDD symptom (137, 142, 143, 246, 255). In light of this evidence, it has been further proposed that dACC may be an initial site of action for NMDA-R antagonists (137), where regional increases in glutamate levels would exert downstream effects on the activity of other ICNs. Moreover, ketamine has been shown to reduce dACC connectivity with frontal and parietal brain areas (135), supporting the hypothesis that reducing elevated connectivity of the dorsal nexus is necessary for reducing depressive symptomatology (78). Specifically, Chen et al. (135) reported that decreases in suicidal thinking after 0.5 mg/kg ketamine infusions, but not 0.2 mg/kg, were associated with diminished left dACC-right sgACC connectivity. Another MEG study by Gilbert et al. that employed dynamic causal modeling demonstrated that dACC-AI connectivity was associated with treatment response (140). Other neuroimaging studies have additionally found that ketamine normalizes SN connectivity dysfunctions related to depression (131, 138, 158), although most of these studies focused on regions of interest that overlapped with other ICNs.

### Frontocerebellar Sensorimotor Network

The SMN comprises the core nodes within the primary motor and somatosensory cortices, which extend to the supplementary motor area (SMA) and subcortical structures, including TH, basal ganglia, sensorimotor nuclei of the brainstem, and cerebellum (256–259). The SMN functions as the brain's transducer, executing reactions and externally directed behaviors in response to incoming inputs. The SMN closely coordinates with other ICNs and has been implicated in several functions associated with error detection, motor planning and initiation, motor inhibition, the subjective urge to move, experience of bodily awareness and pain, and the fine-tuning of cognitive and executive functions (260–264). Neuroimaging studies have indicated that nodes of the SMN display reduced activity (265, 266) and within-network connectivity (86, 88, 158, 267, 268) in MDD patients, which has been further supported by a recent mega-analysis (269). Sensorimotor interventions, such as music, light, tone, and physical exercise, are also well-known to modulate depressive symptoms (270).

Emerging studies have been exploring the association between glutamatergic therapies and SMN functioning, although this comprises a relatively new investigational domain in the field of ICN biomarkers. SMN connectivity has been shown to be capable of predicting antidepressant response to ketamine (157, 158, 212). TRD remitters to ketamine show a significantly elevated connectivity between the cerebellum and basal ganglia at baseline relative to HC, with decreased connectivity following a course of serial ketamine infusions (158). In the most recent study, Loureiro et al. (145) showed that ketamine decreases the within-network connectivity between the cerebellum and other SMN nodes in MDD during a Go/NoGo task in remitters only, and single ketamine infusions have been shown to modulate frontocerebellar loops in MDD patients (153, 271, 272). For instance, Abdallah et al. have shown that ketamine reduces the GBC of the cerebellum (131). Additionally, BOLD activation in SMA has also been suggested to be a viable marker of ketamine treatment, where a decrease in SMA BOLD response at baseline predicted a more favorable treatment response (157). During a response inhibition Go/NoGo task, remitters to ketamine showed a lower pre-treatment SMA BOLD response at baseline relative to non-remitters, which was increased after serial ketamine infusions (157). A single dose of subanesthetic ketamine also appears to increase glucose metabolism in SMA (143), which predicts treatment response (255). A recent GBC study (212) has further shown that reduced within-network SMN connectivity was a robust and reproducible CFP of antidepressant response.

### Frontovagal Central Autonomic Network

The function of the autonomic nervous system is greatly disturbed in MDD, manifested in an overall higher heart rate and lower heart-rate variability that are seen in depressed patients relative to HC (273). Previous research has emphasized the association between depression and cardiovascular disease (274), and sleep quality, sexual functioning, and appetite are routinely assessed in patients as diagnostic features of MDD. Moreover, vagus nerve stimulation (VNS) is an emerging treatment for chronic depression, where electrical pulses delivered to the vagus nerve transmit signals to the areas of the brain that regulate affect and cognition (275). All this evidence indicates an overlap between the heart-brain axis and canonical ICNs implicated in depression (61, 89). The CAN is a relatively “new” ICN that is proposed to be incorporated into the network model of depression, where its hyperactivity might be associated with increased responsivity to stress as well as the general reactivity of the autonomic nervous system to central nervous system perturbations in the domains of affect and cognition.

The CAN is an intricate hierarchical ICN that spans the spinal cord, brainstem, and forebrain (89). HPT is believed to be the core node within the CAN, which projects to the AMYG, insula, and brainstem nuclei responsible for the physiological regulation of visceral organ systems (61, 89). These include the nucleus tractus solitarius, parabrachial nucleus of dorsolateral pons, and ventrolateral medulla, all implicated in the immediate reflexive control of respiration, circulation micturition, and gastrointestinal function. The periaqueductal gray of the midbrain, which integrates autonomic control, behavioral responses to stress and sleep, and pain modulation, is also involved.

The connectivity of the CAN is understudied in humans due to the limited sensitivity and spatial resolution of conventional neuroimaging, as well as the lack of atlases that map deep nuclei of the brainstem and HPT. High-sensitivity and high spatial resolution 7T fMRI, paired with the development of *in vivo* probabilistic atlases, are needed to characterize the structural ad functional connectome of the CAN nodes, and several emerging studies have attempted to do so (276–281). Physiologically, ketamine is known as a sympathomimetic, increasing arterial blood pressure, heart rate, and respiration through direct stimulation of neural structures within the CAN (282, 283). Ketamine is also known to activate subcortical wake-promoting nuclei of the HPT (284), promote the state of arousal (285, 286), increase thalamic metabolism (287), and modulate cholinergic and noradrenergic neurotransmission (285, 288). Moreover, it has a direct effect on the hypothalamic-pituitary-adrenal axis activity and proinflammatory cytokine production, possibly exerting its antidepressant properties through anti-inflammatory pathways (289, 290). To date, however, there are no neuroimaging studies that have attempted to examine the effect of a glutamatergic intervention on the CAN activity or functional connectivity, although preclinical evidence (291) supporting the direct involvement of glutamate in the hypothalamic-pituitary-adrenal axis stress response possibly suggests that glutamate levels in the synapses are positively correlated with the activity and connectivity within the CAN nodes.

### Between-Network Connectivity

**Figure 5:**
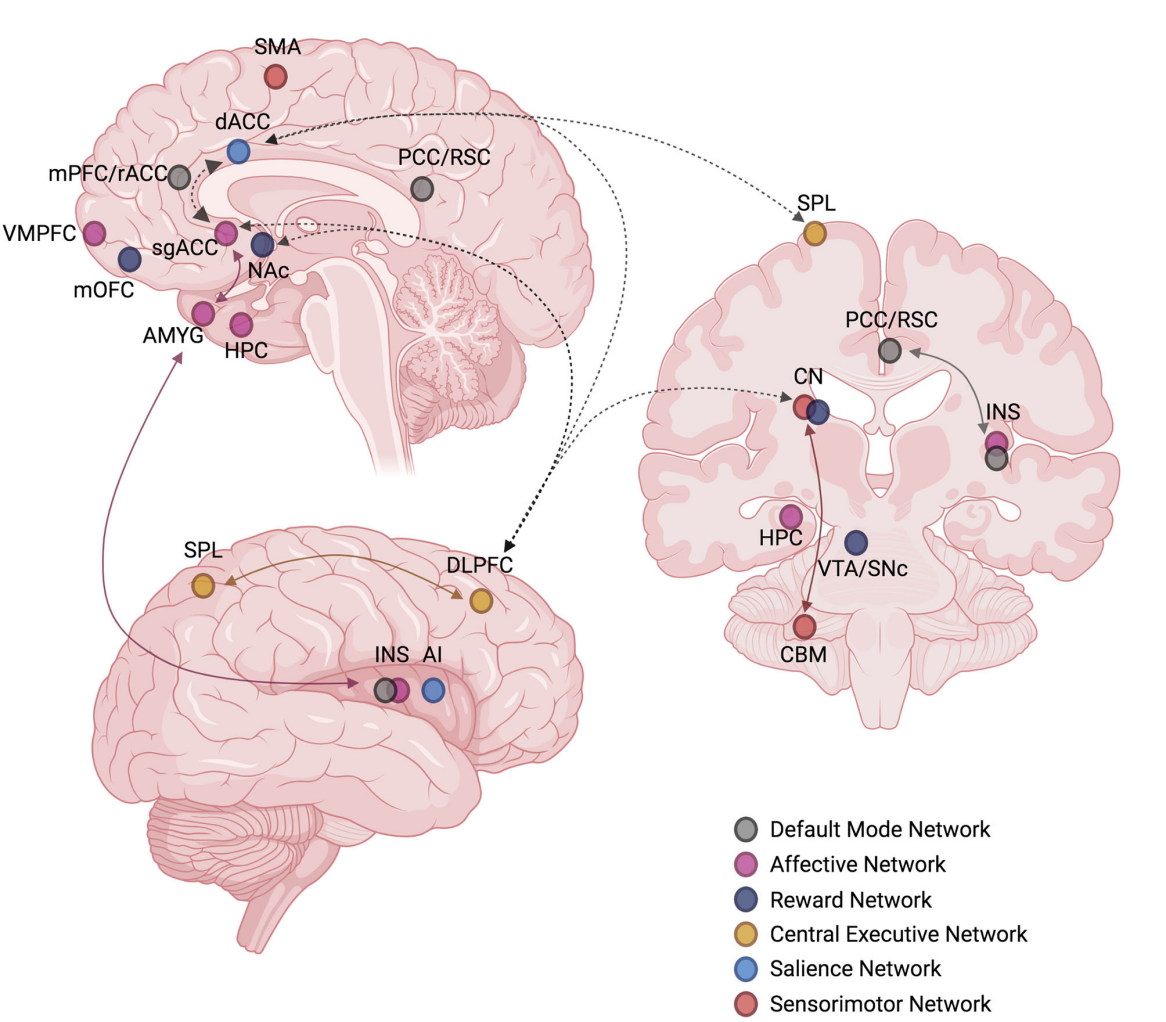
Commonly reproduced intrinsic connectivity network (ICN) nodes and edges targeted by ketamine or implicated in predicting rapid antidepressant treatment response to ketamine. Solid colored arrows indicate within-network connectivity, dashed black arrows indicate between-network connectivity. The laterality of nodes and edges is not considered. Created with BioRender.com, RRID:SCR_018361. AI, anterior insula; AMYG, amygdala; CBM, cerebellum; CN, caudate nucleus; dACC, dorsal anterior cingulate cortex; DLPFC, dorsolateral prefrontal cortex; HPC, hippocampus; INS, insula; mOFC, medial orbitofrontal cortex; mPFC, medial prefrontal cortex; NAc, nucleus accumbens; PCC, posterior cingulate cortex; rACC, rostral anterior cingulate cortex; RSC, retrosplenial cortex; sgACC, subgenual anterior cingulate cortex; SMA, supplementary motor area; SNc, substantia nigra pars compacta; SPL, superior parietal lobule; VMPFC, ventromedial prefrontal cortex; VTA, ventral tegmental area.

It is worth noting that biomarkers of rapid antidepressant response reported by many studies do not perfectly align with only one particular ICN. Many studies have emphasized that glutamatergic interventions target the activity and connectivity of nodes belonging to several ICNs and that certain nodes do, in fact, overlap across multiple ICNs (Figure 5). As such, interconnectivity between major ICNs represents a promising candidate biomarker of rapid response to glutamate-mediating therapeutics that neuroimaging studies have actively explored. In particular, connectivity between the AN and DMN (161), RN and DMN (166), AN and RN (147, 149), RN and CEN (147), DMN and CEN (138), DMN and SN (138), SN and SMN (158), CEN and SMN (145, 212), AN and CEN (165), and AN and SN (165) have all been shown to be modulated by ketamine.

For example, Siegel et el. (161) have shown that ketamine reduces bilateral sgACC connectivity with the DMN. Capitalizing on these findings, Wang et al. further showed that ketamine responders displayed enhanced resting-state connectivity at baseline between the DMN hubs and lateral habenula (LHb), where ketamine-induced downregulation of aberrant LHb connectivity with parts of the DMN might have been attributed to a neural mechanism of ketamine antidepressant effects (166). Murrough et al. (149) documented diminished activation of the right caudate nucleus during the presentation of negatively valenced emotional stimuli in TRD participants, which was normalized following ketamine administration. Resting-state connectivity of the right caudate also predicted antidepressant response, which suggests that the caudate nucleus might be specifically targeted by ketamine. Evans et al. (138) showed that ketamine normalizes the connectivity of the DMN's PCC node with the nodes of the CEN and SN. In particular, 48 h post-ketamine infusion, MDD patients showed an increase in insula-DMN connectivity, suggesting an improved ability to process external stimuli. Furthermore, serial ketamine administration appears to reduce the connectivity of the SN with the cerebellum, a node within the SMN (158), and remitters to ketamine showed a significantly elevated SN-cerebellum connectivity in comparison to HC at baseline. A study by Vasavada et al. (165) explored the effects of four serial ketamine infusions on the connectivity of the AN nodes, notably the AMYG and HPC, with the nodes of the DMN, SN, and CEN among MDD participants and HC. At 24 h post-ketamine, they reported decreased AMYG-left CEN connectivity and increased right HPC-left CEN negative connectivity, with the latter predicting improvements in anhedonia. At 24–48 h post-administration, ketamine increased the AMYG-CEN and right HPC-left CEN negative connectivity, while decreasing the left AMYG-SN connectivity in parallel.

## Intrinsic Connectivity Network Biomarkers and Alternative Glutamate-Mediating Interventions

The efficacy, feasibility, safety, and tolerability of alternative glutamate-mediating interventions have been assessed in numerous MDD clinical trials. Despite these efforts, the ICN biomarker signatures of rapid antidepressant response to these compounds have not yet been identified. At present, there is a minimal number of published peer-reviewed neuroimaging studies that have examined connectivity markers among MDD participants treated with these glutamatergic compounds, although several research groups have incorporated neuroimaging protocols into their active trials and have presented preliminary results at scientific meetings and conferences. The widespread interest in the glutamate hypothesis of depression and the existing evidence showcasing that ketamine successfully targets disrupted brain connectivity in depression makes it incredibly likely that the clinical and academic community can expect novel exciting developments in this area in the next decade.

### Memantine

Memantine is a non-competitive NMDA-R antagonist of low-to-moderate affinity that is used for the treatment of cognitive symptoms of dementia (292). The key advantage of memantine over ketamine is that it does not cause psychomimetic effects at therapeutic doses (293), which spurred a hypothesis that it potentially may be a better alternative to ketamine for the treatment of depression. In the context of MDD, memantine has been studied mainly as an augmentation for conventional antidepressants, although memantine failed to improve their treatment efficacy for depressive symptoms (294). Nevertheless, in a study (167) with 26 MDD older adults (60+ years) with subjective memory complaints who were randomized to receive escitalopram/memantine vs. escitalopram/placebo, at 3 months after treatment initiation, the relationship between increased connectivity within posterior and lateral nodes of the DMN and improvement in depression severity was further enhanced by memantine than with placebo, which suggests that addition of memantine to monoaminergic pharmacotherapy improves the engagement with the neurocircuitry in geriatric depression. Structural studies (295, 296) have further shown that memantine contributes to increases in gray matter volume and cortical thickness in the regions of the right OFC and left middle and inferior temporal lobes, as well as to regional changes in white matter integrity within the tracts of the bilateral anterior and posterior limbs of the internal capsule, bilateral inferior fronto-occipital fasciculus, and right superior fronto-occipital fasciculus. White matter integrity changes were also predictive of antidepressant response in TRD older adults who received combined escitalopram and memantine but not in those who received combined escitalopram and placebo (296).

### D-Cycloserine/NRX-101

D-cycloserine (DCS), or NRX-101, is a partial NMDA-R agonist at a glycine modulatory site that acts as an NMDA-R antagonist at high doses (297). Its antidepressant properties have been demonstrated in a clinical trial setting among 26 TRD adults (298), although no replication or biomarker studies have been published since then. Recently, however, DCS has been tested as an augmentation treatment and has been shown to maintain antidepressant and antisuicidal effects of ketamine in TRD patients, although with no efficacy as a monotherapy (299). A new line of neurophysiological research is under development, where DCS administration is paired with neurostimulation techniques, such as rTMS, to investigate its effects on synaptic plasticity in the nodes of the SMN among depressed patients (168, 300, 301). A recent study by Cole et al. (168) has shown that DCS normalizes stimulus-response curves in the motor cortex of MDD patients 24 h after post-intermittent theta-burst stimulation-rTMS, which suggests that DCS and its action on NMDA-Rs may rearrange the connectivity within the SMA.

### Nitrous Oxide

Nitrous oxide (N_2_O), also known as the laughing gas, has been used as an anesthetic for almost a century. Its primary mechanism of action is believed to be the NMDA-R antagonism, although research indicates that AMPA-R, kainate receptors, nicotinic acetylcholine receptors, calcium and potassium channels, and the opioid system contribute to its antidepressant effects (302–304). Several clinical trials (305–307) have shown that N_2_O is potentially effective for reducing depression symptoms, and several Phase II/III trials are currently underway. Since reductions in fMRI functional connectivity in the regions of the dorsal nexus, DMN, and AN have already been demonstrated with NMDA-R antagonists, such as ketamine, it has been proposed that similar changes may occur with N_2_O treatment (308).

### 4-Chlorokynurenine/AV-101

4-chlorokynurenine (4-Cl-KYN), or AV-101, is a derivative of 7-chlorokynurenic acid—a well-known NMDA-R glycine site antagonist (309). 4-Cl-KYN has been tested as a 14-day monotherapy for TRD in a crossover Phase II trial (169), but the 4-Cl-KYN treatment arm did not show a significant improvement in depressive scores relative to the placebo arm. Biomarker outcome measures also included the assessment of 7T ^1^H-MRS brain glutamate levels and resting-state fMRI connectivity, and no difference was observed between the two treatment arms for any of these biological indices.

### Lanicemine/AZD6765

Lanicemine, also known as AZD6765, is a low-affinity and low-trapping NMDA-R antagonist that possesses properties similar to ketamine. Despite its favorable pharmacokinetic properties, the clinical trials (310–313) have failed to demonstrate significant antidepressant effects. In a comparative study on unmedicated MDD patients randomized to receive ketamine, lanicemine, or placebo, ketamine significantly increased the GBC of the DLPFC, DMPFC, and mPFC during infusion and at 24 h post-treatment, while lanicemine failed to do so (133). This suggested that ketamine and lanicemine have different neurobiological properties despite the shared pharmacodynamic profile. Lanicemine has been compared with ketamine in its ability to downregulate the activity of the sgACC in unmedicated MDD adults, and both compounds were found to increase the BOLD response in the sgACC as well as the TH after a single infusion (137).

## Intrinsic Connectivity Networks and Slow-Onset Monoaminergic Antidepressants: Is there a Difference?

Slow-onset monoaminergic pharmacotherapies and their relationship to brain connectivity changes is a major research topic of interest, given that, in the clinical setting, monoamines are still prescribed as the first-line treatment for MDD. As of now, the need for robust and reproducible biomarkers of monoamine antidepressant response is apparent since those potentially have a greater prospective clinical utility. Conventional antidepressants have, in fact, demonstrated promise in terms of mitigating brain network connectivity abnormalities among MDD participants. For instance, fMRI studies have reported changes in the DMN, AN, RN, and CEN functional connectivity following antidepressant treatment with SSRIs or serotonin-norepinephrine reuptake inhibitors (SNRIs) (23, 30, 32, 38, 314–318). Further, research has suggested that baseline structural and functional connectivity of the DMN, SN, AN (sgACC, HPC, AMYG), and CEN (DLPFC) may predict antidepressant treatment response (23, 38). In particular, stronger posterior-anterior DMN connectivity prior to treatment, as well as increased connectivity within this network generally, have been found to correlate with the treatment response following monoaminergic pharmacotherapy (32, 314, 318). Further, greater RN-DMN pre-treatment functional connectivity, specifically between the NAc and rostral ACC, has been found to be associated with bupropion treatment response (319). With respect to the AN and SMN, decreased AMYG functional connectivity with the left SMA and right precentral gyrus, as well as increased AMYG functional connectivity with the right central opercular cortex, have been found to reflect a favorable response to SSRI treatment (320). Previous research has also found that areas of the CEN with low functional connectivity, such as the DLPFC, are associated with a greater response to sertraline (321). In contrast, decreased functional connectivity of the right insula, a node of the SN, has been linked with insufficient antidepressant response (322). Between-network connectivity has also been suggested to be a predictor of response to pharmacotherapy. A large study on patterns of functional connectivity and sertraline treatment outcomes in MDD found that, in general, greater between-network connectivity predicted a better response to sertraline (314). In addition, greater hippocampal connectivity to various networks (e.g., HPC-DAN) and decreased connectivity with the SN was associated with a better placebo response (314). Taken together, these findings suggest that connectivity biomarkers in MDD are complementary in predicting response to slow-onset monoaminergic pharmacotherapies.

Despite these advances, the therapeutic time lag and inadequate response rates that are associated with monoamine therapies make them a suboptimal treatment option for MDD (323), and novel glutamatergic treatment options might be a better alternative. The hypothesis of monoamine deficiency lacks a solid evidence base, but since a variety of treatment interventions of different forms and styles are efficacious for depression, including conventional and non-conventional pharmacotherapy, brain stimulation, psychotherapy, it is reasonable to infer that these modalities would act on a converging final common pathway that underlies antidepressant response (7). Neuroplasticity, or structural and functional processes related to growth, maturation, apoptosis, and communication of neurons, as well as their ability to change in response to the environment, is thought to be the “final common pathway” of antidepressant response that can be targeted by multiple treatment interventions.

Both monoaminergic and glutamatergic antidepressants improve morphological and functional neuroplasticity. In fact, mechanisms of action of monoaminergic and glutamatergic antidepressants are thought to converge at the synaptic connectivity level, where both modalities target neurotrophic factors leading to neuroplasticity changes in synaptic connectivity and neural wiring (45, 212, 324). The key molecular pathways where the two types of antidepressants converge include the reduction of the depolarization-evoked release of presynaptic glutamate, which mitigates the effect of excitotoxicity and stimulates neurogenesis and synaptic strength, the enhancement of AMPA-R promoted by the inactivation of NMDA-R, the induction of long-term potentiation processes, and the release of growth factors, such as BDNF [for review, see (7)]. These molecular mechanisms subsequently result in changes in neurocircuitry and network properties, leading to behavioral manifestations of antidepressant response (45). This is supported by recent advances in neuroimaging, where connectivity studies have shown a high association between glutamatergic and monoaminergic CFPs in the MDD population and where a unique antidepressant CFP identified for ketamine predicted treatment response to monoaminergic pharmacotherapy (151, 212).

However, monoamines mainly act on a serotonin transporter, which only indirectly regulates the activity of glutamatergic receptors. While it plays an important role in modulating neuroplasticity, this pathway is much weaker, slower, and less efficient in comparison to the direct modulation of ionotropic glutamate receptors exerted by ketamine and other interventions (7). Glutamate is the brain's major excitatory neurotransmitter, and all brain functions related to cognition and emotions are ultimately mediated by the interplay between excitatory glutamatergic transmission and inhibitory GABAergic transmission (16). The ICNs discussed in this review comprise glutamatergic connections by more than 80%, while monoaminergic connections account for a significantly lower proportion. Ketamine and other related interventions would take a faster and shorter route to promote neuroplasticity and induce network-level remodeling, leading not only to a faster antidepressant response at the behavioral level but also to more widespread and long-term neurobiological changes.

## Future Directions for Glutamate-Based Therapeutics

Given that MDD is marked by limited responsivity to conventional pharmacotherapy and psychotherapy (1, 11), biomarkers of response to newly emerging alternative treatments are in high demand. Advances in functional neuroimaging have enabled the identification of neural correlates of rapid antidepressant response with robustness and specificity, and, as evident from this review, they will continue serving as a valuable tool for the future generation of biomarker studies. While the field of glutamatergic treatments for mood disorders greatly benefits from emerging functional neuroimaging, genetic, and neurophysiological studies, structural brain imaging research remains limited. Likewise, the functional neuroplasticity hypothesis that is currently the leading model in the field needs to be accompanied by specific evidence of structural plasticity, which provides opportunities for reverse translation to animal models. In clinical research, conversely, supplementing the field with diffusion-based neuroimaging and longitudinal volumetric data can be of major benefit to the future generation of biomarker studies that will strive to elucidate where and how glutamatergic interventions act upon distinct brain systems. Integrating structural and functional data will also enhance the accuracy and power of predictive models since models constructed through multiple integrated modalities tend to perform better than those trained using a single modality (325).

This review proposes a neuroanatomical framework for studying glutamatergic ICNs in depression. While this approach may reflect a reliance on a limited number of nodes and edges that are selected *a priori*, it can improve the precision and specificity of glutamate-based therapeutics and put research findings into medical perspective, bringing them closer to clinical practice and prospective use for diagnostic and treatment purposes. The ICN framework, in particular, is a valid and reproducible approach inspired by the principles of graph theory that mirrors the hierarchical organization of the brain's intrinsic architecture (182, 183, 188). Neuroimaging studies striving to describe the systems-level effects of glutamatergic treatments may greatly benefit from the characterization of ICNs provided here, which will aid in enabling the exploration of connectomic signatures of rapid antidepressant effects in a harmonized fashion. Correspondingly, the ICN theoretical model can be applied across brain imaging studies for the purposes of repeatability and reproducibility, as well as systematized interpretation of findings. This may ensure faster and more efficient progress in the development of effective glutamate-based therapies for mood disorders.

Existing literature reviews in the field of glutamatergic ICNs support the notion that glutamate-based therapies act on several brain networks, leading to a rapid antidepressant response (38, 326, 327). As discussed by Alario and Niciu (326), further research exploring how glutamate-based therapies for depression, such as ketamine, have long-lasting effects with short-term connectivity changes is needed. The promising findings that exist to date suggest the importance of coordinating the timing of study and neuroimaging procedures in a way that is physiologically appropriate to capture acute vs. delayed effects of glutamate-based therapies within affected ICNs (326). Future research may also employ long-term follow-ups to generate an understanding of how such delayed effects occur at a physiological level and provide insight into sustainable long-term changes in brain connectivity following therapeutics that target the glutamate system.

Furthermore, the biomarker studies reviewed here are characterized by a notable variability in employed neuroimaging procedures and analysis methods (Table 3). Other authors have also raised this issue. For instance, Kotoula et al. (327) points to a discrepancy between ways of measuring brain connectivity across healthy and depressed samples, as well as between ways of measuring acute and delayed effects of treatment interventions, including ketamine. This makes the interpretation of ICN findings challenging, and the use of different analysis pipelines within a single study may act as a potential confound. Further, the tendency to use small neuroimaging sample sizes with such novel therapeutics may also prevent a true understanding of how glutamate-based therapies act on various brain networks. Future research should use harmonized neuroimaging procedures and analyses and larger MDD and HC sample sizes across studies and sites.

Nevertheless, it seems evident that ICN changes induced by ketamine and other glutamatergic treatments comprise both the elevation and the reduction in functional connectivity and nodal strength. The prevailing theoretical model behind these mechanisms is the modulation of functional neuroplasticity, where glutamatergic compounds lead to rapid alterations in synaptic connectivity. As noted by Abdallah et al. (212), it seems likely that both increases and decreases in synaptic connectivity are needed to induce antidepressant effects. However, how changes in neuroplasticity at a molecular and cellular level translate into system-level changes in ICNs remains largely unknown, both in the context of glutamatergic modulation and in the context of depression. Marrying the modalities of genetics, cellular electrophysiology, neuroimaging, and behavioral markers has the potential to bridge this translational gap, and trials with this aim that explore serotonergic antidepressant response are already underway (328, 329). For glutamate-based therapeutics, this should be the focus of prospective research in the next decade, where neuroimaging would be used as a “tool in the toolkit” (23) and not as the only method for drawing conclusions about complex biological processes that drive network changes.

## Conclusions

Through this review, we have summarized advances in the understanding of how glutamate-modulating interventions impact ICNs of the human brain. The reviewed evidence suggests that there are favorable prospects in using glutamate-modulating interventions to target human ICNs that are implicated in depression. Further, we have identified relevant biomarkers of rapid antidepressant response, such as altered functional connectivity of limbic, cognitive, and executive nodes, to aid in understanding and rationalizing the use of glutamate-based therapeutics as a neurobiologically sound alternative to existing slow-acting treatment options for depression. As research in this field continues to emerge, it may become evident how structural and functional changes within implicated ICNs translate into improvements in clinical and behavioral manifestations of depression. Finally, we have contrasted the connectomic theoretical principles underlying slow-onset monoaminergic pharmacotherapies with those of glutamatergic interventions, highlighting the convergence of their mechanisms of action on processes that regulate and drive neuroplasticity. Such preliminary neurobiological evidence supports the use of glutamate-based pharmacotherapies for disorders of brain dysconnectivity, such as depression.

## Author Contributions

ID, KD, and VB co-designed the theoretical framework for this article. ID and VB conceptualized the manuscript. ID performed the literature search and analysis, drafted the initial version of the manuscript, created figures and tables, and participated in subsequent review and editing. VT contributed to manuscript writing, performed literature investigation, assisted with the preparation of tables, and participated in review and editing. SK contributed valuable ideas to the conception of the article and assisted with review and editing. KD contributed valuable ideas to the conception of the article, provided writing supervision, and assisted with review and editing. VB provided primary supervision for creating this review and critically revised the manuscript. All authors approved the final version of this article.

## Conflict of Interest

SK has received honoraria or research funds from Abbott, Alkermes, Allergan, Boehringer Ingelheim, Brain Canada, Canadian Institutes of Health Research, Janssen, Lundbeck, Lundbeck Institute, Ontario Brain Institute, Ontario Research Fund, Otsuka, Pfizer, Servier, Sunovion, Sun Pharmaceuticals, and holds stock in Field Trip Health. KD was supported by an Academic Scholar Award from the University of Toronto, Department of Psychiatry. VB is supported by an Academic Scholar Award from the University of Toronto, Department of Psychiatry, and has received research support from the Canadian Institutes of Health Research, Brain & Behavior Foundation, Ministry of Health Innovation Funds, Royal College of Physicians and Surgeons of Canada, Department of National Defense (Government of Canada), New Frontiers in Research Fund, Associated Medical Services Healthcare Inc, Novartis, and from Roche Canada for an investigator-initiated trial. The remaining authors declare that the research was conducted in the absence of any commercial or financial relationships that could be construed as a potential conflict of interest.

## Publisher's Note

All claims expressed in this article are solely those of the authors and do not necessarily represent those of their affiliated organizations, or those of the publisher, the editors and the reviewers. Any product that may be evaluated in this article, or claim that may be made by its manufacturer, is not guaranteed or endorsed by the publisher.

## Abbreviations

^18^F-FDG: Fluorodeoxyglucose
^1^H-MRS: Proton Magnetic Resonance Spectroscopy
4-CI-KYN: 4-chlorokynurenine
ACC: Anterior cingulate cortex
AI: Anterior insula
AMPA-R: α-amino-3-hydroxy-5-methyl-4-isoxazolepropionic acid receptors
AMYG: Amygdala
AN: Affective Network
BDNF: Brain-derived neurotrophic factor
*BG (figures/tables only)*: *Basal ganglia*
*BS (figures/tables only)*: *Brainstem*
BOLD: Blood-oxygen-level-dependent
CaMKII: Calcium/Calmodulin-Dependent Protein Kinase II
*CN (figures/tables only)*: *Caudate nucleus*
CAN: Central Autonomic Network
CCN: Cognitive Control Network
CEN: Central Executive Network
*CBM (figures/tables only)*: *Cerebellum*
CFP: Connectome fingerprint
CPM: Connectome-based predictive modeling
dACC: Dorsal anterior cingulate cortex
DAN: Dorsal Attention Network
DCS: D-cycloserine
DLPFC: Dorsolateral prefrontal cortex
DMN: Default Mode Network
DMPFC: Dorsomedial prefrontal cortex
ECT: Electroconvulsive therapy
eEF2K: Eukaryotic Elongation Factor 2 Kinase
EEG: Electroencephalography
*FEF (figures/tables only)*: *Frontal eye fields*
fMRI: Functional Magnetic Resonance Imaging
GABA: Gamma-aminobutyric acid
GBC: Global Brain Connectivity
GBCr: Global Brain Connectivity with global signal regression
GLX: Glutamate + Glutamine
GM: Gray Matter
*GP (figures/tables only)*: *Globus pallidus*
GPu SC: Globus pallidus-Putamen Subcortical
GSK3: Glycogen synthase kinase-3
HC: Healthy controls
HPC: Hippocampus
HPT: Hypothalamus
ICNs: Intrinsic Connectivity Networks
IPL: Inferior parietal lobule
*INS (figures/tables only)*: *Insula*
*IPS (figures/tables only)*: *Intraparietal sulcus*
LHb: Lateral habenula
*LTL (figures/tables only), Lateral temporal lobe; M1 (figures/tables only)*: *Primary motor cortex*
MDD: Major Depressive Disorder
MeCP2: Methyl-CpG-binding protein 2
MEG: Magnetoencephalography
mGluRs: Metabotropic glutamate receptors
mOFC: Medial orbitofrontal cortex
*MTL (figures/tables only)*: *Medial temporal lobe*
mPFC: Medial prefrontal cortex
mTOR: Mammalian target of rapamycin
N_2_O: Nitrous oxide
NAc: Nucleus accumbens
NMDA-R: N-methyl-D-aspartate receptor
NRS: Network-restricted strength
*NTS (figures/tables only)*: *Nucleus tractus solitarius*
*PBM (figures/tables only)*: *Parabrachial nucleus*
*PAG (figures/tables only)*: *Periaqueductal gray*
PCC: Posterior cingulate cortex
PCu: Precuneus
PET: Positron Emission Tomography
PFC: Prefrontal cortex
pgACC: Pregenual anterior cingulate cortex
*Pu (figures/tables only)*: *Putamen*
rACC: Rostral anterior cingulate cortex
RN: Reward Network
*RSC (figures/tables only)*: *Retrosplenial cortex*
rTMS: Repetitive transcranial magnetic stimulation
*S1*: *(figures/tables only), Primary somatosensory cortex;*
*sgACC*: Subgenual anterior cingulate cortex
SMA: Supplementary motor area
SMN: Sensorimotor Network
SN: Salience Network
SNc: Substantia nigra pars compacta
SNRI: Serotonin-Norepinephrine Reuptake Inhibitor
SPL: Superior parietal lobule
SSRI: Serotonin Selective Reuptake Inhibitor
TH: Thalamus
TPJ: Temporoparietal junction
TRD: Treatment-Resistant Depression
*vACC*: *(figures/tables only), Ventral anterior cingulate cortex*
VAN: Ventral Attention Network
*VLM (figures/tables only)*: Ventrolateral medulla
VMPFC: Ventromedial prefrontal cortex
VTA: Ventral tegmental area.
